# Patterns of Mental Health Service Utilisation: A Population-Based Linkage of Over 17 Years of Health Administrative Records

**DOI:** 10.1007/s10597-024-01300-8

**Published:** 2024-06-12

**Authors:** Crystal Man Ying Lee, Kevin Chai, Peter M. McEvoy, Kyran Graham-Schmidt, Daniel Rock, Kim S. Betts, Justin Manuel, Mathew Coleman, Shiv Meka, Rosa Alati, Suzanne Robinson

**Affiliations:** 1 School of Population Health, Curtin University, Perth, WA Australia; 2 Centre for Clinical Interventions, North Metropolitan Health Service, Perth, WA Australia; 3 Department of Health, Perth, WA Australia; 4 WA Primary Health Alliance, Perth, WA Australia; 5 Discipline of Psychiatry, Medical School, University of Western Australia, Perth, WA Australia; 6 Faculty of Health, University of Canberra, Canberra, ACT Australia; 7 WA Country Health Service, Perth, WA Australia; 8 WA Country Health Service, Albany, WA Australia; 9 East Metropolitan Health Service, Perth, WA Australia; 10 Deakin Health Economics, Deakin University, Melbourne, VIC Australia

**Keywords:** Community mental health, Emergency department, Inpatient psychiatry, Health service research

## Abstract

A cross-sectoral partnership was formed in 2021 in support of the recommendations in an audit on access to state-funded mental health services. In this first paper, we aimed to describe the demographic and service utilisation of adults with a mental health diagnosis in the Western Australian state-funded health system from 2005 to 2021. Inpatient, emergency department, specialised (ambulatory) community mental health service, and death records were linked in individuals aged ≥ 18 years with a mental health diagnosis in Western Australia. Altogether, 392,238 individuals with at least one mental health service contact between 1st January 2005 and 31st December 2021 were included for analysis. Females, Aboriginal and/or Torres Strait Islander people, and those who lived outside major cities or in the most disadvantaged areas were more likely to access state-funded mental health services. While the number of individuals who accessed community mental health services increased over time (from 28,769 in 2005 to 50,690 in 2021), the percentage increase relative to 2005 was notably greater for emergency department attendances (127% for emergency department; 76% for community; and 63% for inpatient). Conditions that contributed to the increase for emergency department were mainly alcohol disorder, reaction to severe stress and adjustment disorders, and anxiety disorders. Sex differences were observed between conditions. The pattern of access increased for emergency department and the community plus emergency department combination. This study confirmed that the patterns of access of state-funded mental health services have changed markedly over time and the potential drivers underlying these changes warrant further investigation.

**Supplementary Information:**

The online version contains supplementary material available at 10.1007/s10597-024-01300-8.

## Introduction

In 2020–21, 8.6 million (43.7%) Australians aged 16–85 years had experienced a mental disorder in their lifetime and almost half of whom (4.2 million or 48.9%) had experienced a mental disorder in the past 12 months (Australian Bureau of Statistics (ABS), 2022). These figures represent a 17.8% (lifetime) and 31.3% (12-month) increase from the previous National Survey of Mental Health and Wellbeing in 2007 (ABS, 2008). Despite COVID-19 restrictions in some Australian jurisdictions during the 2020–21 survey period, the proportion of individuals with mental disorders in the past 12 months who had at least one consultation with a health professional for their mental health increased from 34.9% in 2007 to 47.1% in 2020–21. For those with lifetime mental disorders but without symptoms in the past 12 months, mental health service use increased from 9.2% in 2007 to 13.9% in 2020–21 (ABS, 2008; ABS, 2022). Increasing our understanding of who is accessing services and pathways through the mental health system is critical to identifying opportunities for service efficiencies that will better meet consumers’ needs and improve health outcomes.

Western Australia (WA) is the largest state in Australia covering 32.9% of the total land area and 10.8% of the total population (ABS, 2023a). Approximately 78.6% of the population reside in the only major city in the state, which covers only 0.1% of the state’s land area. In 2017–18, 441,300 (17.8%) West Australians had a mental or behavioural condition, an increase from 358,100 (14.6%) individuals in 2014–15 (ABS, 2018). In accordance with the Australian mental health system, mental health care delivery in WA includes a mix of Commonwealth government, state government and individually funded services (Australian Institute of Health and Welfare (AIHW), 2023a). For example, consultations with primary care providers and private specialists and prescriptions of psychotropics are subsidised by the Commonwealth government while treatments received in public hospitals and community mental health services are covered by state governments. Out-of-pocket expenses and services provided by the private sector are borne by individuals.

In 2019, an audit on access to state-funded adult mental health services was conducted in WA (Office of the Auditor General Western Australia, 2019). The report indicated data have traditionally been used to quantify health services activity rather than the healthcare access patterns of individuals with mental health conditions and, consequently, recommended the analysis of patient pathways across state mental health services to (1) inform service reconfiguration where required and (2) help prioritise initiatives in the Western Australian Mental Health and Drug Services Plans. In support of the recommendation, a cross-sectoral partnership between Curtin University, Digital Health Cooperative Research Centre, Department of Health WA, WA Country Health Service, and WA Primary Health Alliance was formed in 2021. The overarching aim of the partnership is to use linked health administrative records in WA to support population health planning and clinical management of individuals with mental or behavioural disorders by providing answers to industry driven research questions.

In this first paper, we aimed to describe the linked data, and begin to characterise the state-funded mental health service adult user population in WA from 2005 to 2021 and report on the pattern of mental health service use during this period.

## Methods

### Data Source

This is a population-based mental health linkage project of health administrative records in WA with the aim of investigating journeys of mental health patients through the healthcare system. The linked data were sourced from administrative health data systems held by the WA Department of Health and the death register held by the Department of Justice. Linkage was carried out by the Western Australian Data Linkage Branch using probabilistic record linkage methods, which consider errors in the matching of variables rather than requiring an exact match (Hodges et al., 2020). The data collections contain record level data on hospital admissions [Hospital Morbidity Data Collection (HMDC)], emergency department (ED) presentations [Emergency Department Data Collection (EDDC)], community mental health service contacts and mental health assessments [Mental Health Data Collection (MHDC)], and mortality status (Death Register). Details of each data collection are described below. Records of all individuals aged ≥ 18 years were linked if they had at least one (1) recorded entry in the MHDC since 2005; (2) a principal or additional diagnosis for mental and behavioural disorders in the HMDC since 2005; and/or (3) a principal or additional diagnosis for mental and behavioural disorders in the EDDC since 2005. Mental and behavioural disorders were classified according to the International Statistical Classification of Diseases and Related Health Problems, Tenth Revision, Australian Modification (ICD-10-AM) (National Centre for Classification in Health, 2017): F00–99, O99.3, Q86.0, R44–46, T39–52, T58, X60–84, Y87.0, Z00.4, Z03.2, Z04.6, Z09.3, Z13.3, Z59–65, Z71.4–71.5, Z72–73, Z86.4–86.5, Z91.5–91.6, U79 (Table S1). Records were extracted from 1st January 2005 to the most recent record available at the time of extraction (i.e., 30th April 2022 for HMDC; 30th June 2022 for MHDC; 31st August 2022 for EDDC and Death Registration). Altogether, records of 1,102,841 individuals with possible mental and behavioural disorders were available and formed the cohort for this mental health data linkage project.

#### Hospital Morbidity Data Collection

The HMDC includes record level data related to episodes of care of patients admitted to both public and private acute hospitals, both public and private psychiatric hospitals, and private day surgeries in WA (Government of Western Australia Department of Health, 2020a). For each separation (i.e., hospital stay), data are available on patient demographics (i.e., age, sex, Aboriginal and/or Torres Strait Islander status, employment status, marital status, remoteness of residence, socioeconomic area of residence), administrative information (e.g., admission date, separation date (i.e., date of hospital discharge or death), mental health legal status) and clinical information (e.g., principal diagnosis, co-occurring diagnosis, up to 20 additional diagnoses, up to four external causes of injury) (Government of Western Australia Department of Health, 2022a).

#### Emergency Department Data Collection

The EDDC includes record level data related to episodes of care within EDs at public hospitals and major contracted health entities, and emergency services provided in hospitals without an ED in WA (Government of Western Australia Department of Health, 2020b). For each episode of care, data are available on patient demographics, administrative information (e.g., human intent of injury, short stay unit destination on departure, referral source) and clinical information (e.g., principal diagnosis, additional diagnosis) (Government of Western Australia Department of Health, 2022b).

#### Mental Health Data Collection

The MHDC includes the Mental Health Information System and the Mental Health Information Data Collection, which replaced the Mental Health Information System on 1st January 2018 (Government of Western Australia Department of Health, 2020c). The MHDC includes record level data of individuals who have accessed specialised ambulatory (non-admitted) mental health services in the Western Australian public system (community mental health services). For each community mental health service contact, data are available on patient demographics, administrative information (e.g., mental health service program, service contact medium), clinical information (e.g., principal diagnosis at admission and discharge, triage presenting problem), and mental health clinical outcomes assessments (i.e., Health of Nation Outcome Scales (HoNOS) (Wing et al., 1996), HoNOS for elderly people (HoNOS 65 +) (Burns et al., 1999), Kessler Psychological Distress Scale (K10) and K10 plus (Kessler & Mroczek, 1992), Life Skills Profile (Rosen et al., 1989), Resource Utilisation Groups – Activities of Daily Living) (Fries et al., 1994; Government of Western Australia Department of Health, 2022c).

#### Death Registrations

The Death Register includes records of all deaths that occurred in WA.

This project was approved by the Department of Health WA Human Research Ethics Committee and the Curtin University Human Research Ethics Committee. A waiver of consent was sought for the project.

### Study Participants

In this study, individuals were included for analysis if they had at least one mental health service contact in at least one care setting between 1st January 2005 and 31st December 2021. Therefore, individuals with possible mental and behavioural disorders whose health service contacts were all non-mental health related were excluded from this study. Inpatient mental health service contacts were defined as records with an ICD-10-AM code of F00–99 in principal diagnosis or co-occurring diagnosis; R45.81, Z00.4, Z03.2, Z04.6, Z09.3, Z13.3–13.4, Z50.2–50.4, Z54.3, Z86.4, Z91.4–91.5 coded in principal diagnosis; X60–84 coded in external cause of injury; care type as “mental health care”; the episode of care occurred in a designated mental health unit; mental health legal status was not missing; and/or major diagnostic category as “mental diseases and disorders” or “substance use and substance induced organic mental disorders”. Community mental health service contacts included all records in the MHDC except those with service event category as “staff” or “pre-referral”. While EDs do not provide specialised mental health service, ED presentations with a mental health diagnosis are substantial. Therefore, ED mental health service contacts were records with F00–99 coded in principal diagnosis or additional diagnosis; presenting complaint with mental health related hospital specific codes; and/or major diagnostic category as “mental diseases and disorders” or “substance use and substance induced organic mental disorders”.

### Data analysis

#### Patient characteristics

We reported the percentages of individuals on the (1) first contact with state-funded mental health service in any care setting over age 18 years since 2005; (2) first contact within a mental health care setting (i.e., community, ED, inpatient) over age 18 years since 2005; and (3) first recorded relevant mental health conditions over age 18 years since 2005; by sociodemographic characteristics. Mental health conditions were derived by combining ICD-10-AM codes for mental and behavioural disorders (i.e., F00-F99) into 22 categories (Table S2). Therefore, individuals without a recorded F code were not included in the analysis on mental health conditions and those with more than one F code were included in analyses relevant to the conditions associated with the F codes. Socioeconomic status was defined based on the Socio-Economic Indexes for Areas—Index of Relative Socio-Economic Disadvantage (SEIFA-IRSD) scores for local government areas which is a summary measure based on census data that ranks geographic areas across Australia according to their relative socioeconomic disadvantage (ABS, 2023a). The cut-off scores for each SEIFA-IRSD decile obtained from the ABS were used to assign one of ten socioeconomic groups and then collapsed into five groups (i.e., quintile 1 (most disadvantaged), 2, 3, 4, 5 (least disadvantaged)).

#### Mental Health Service Use

We reported the total number of individuals who accessed mental health service at least once within a calendar year from 2005 to 2021, and the percentage change in the number of individuals relative to 2005, by care setting. The year of service contact was based on the contact date for community mental health service and the presentation date for ED mental health service. For inpatient stay with admission date in one calendar year and discharge date in another calendar year, access of inpatient mental health service was counted in every calendar year over the length of stay. In addition, we reported the patterns of access of mental health services for the period 2005–2021 and by year. All analyses were repeated by sex. To reduce potential misclassification of non-mental health related inpatient service, the total number of individuals who accessed inpatient mental health service and the pattern of access were repeated using a stricter definition (i.e., for care outside a designated mental health unit, the inpatient stay was classified as a mental health stay if at least one of the criterion outlined above (i.e., care type, external cause of injury, major diagnostic category, mental health legal status) was met in addition to the diagnosis criteria stated above). Furthermore, the total number of individuals who accessed mental health service by care setting was repeated by mental health conditions. Statistical analyses were performed using Python and SAS 9.4.

## Results

### Patient Characteristics

#### First Mental Health Service Contact

Overall, 392,238 individuals met the inclusion criteria. Of these, 60.0% were aged < 45 years, 53.8% were females, 7.6% were Aboriginal and/or Torres Strait Islanders, 60.5% resided in major cities, and 41.5% resided in the least disadvantaged areas (quintile 5) on their first recorded contact with state-funded mental health service over age 18 years since 2005 (Table 1). Compared to the Western Australian population in 2021 (ABS, 2023a; ABS, 2023b; ABS, 2023c), the cohort was more likely to be younger, female, Aboriginal and/or Torres Strait Islander and to have resided in the most disadvantaged areas but less likely to have lived in major cities on their first mental health service contact in adulthood. In general, the highest percentages of the first mental health service contact occurred in the community setting and the lowest percentages occurred in the inpatient setting (e.g., all individuals: 48.6% for community; 39.8% for ED; 11.7% for inpatient) except for the Aboriginal and/or Torres Strait Islander (42.8% for community; 48.1% for ED; 9.1% for inpatient) and most disadvantaged (42.8% for community; 47.9% for ED; 10.3% for inpatient) subgroups, where the percentages were highest in the ED setting. When stratified by sex, the percentages were higher for ED compared to community in the aged 18–24 years, Aboriginal and/or Torres Strait Islander, very remote areas, and the most disadvantaged (quintile 1) and quintile 4 subgroups for males and in the Aboriginal and/or Torres Strait Islander and the most disadvantaged subgroups for females (Table S3).

**Table 1:** Characteristics of individuals on their first recorded contact with the Western Australian state funded mental health service over age 18 years since 2005

	Western Australian population in 2021^a^ N (%)	Current study n (%)	Mental health care setting at first contact		
			Community (%)	Emergency department (%)	Inpatient (%)
All (age ≥ 18 years)	2,122,191	392,238	48.6	39.8	11.7
Age					
18–24 years	227,916 (10.7)	89,283 (22.8)	47.2	45.1	7.7
25–34 years	391,233 (18.4)	83,025 (21.2)	53.8	38.3	7.9
35–44 years	391,912 (18.5)	62,997 (16.1)	51.4	37.9	10.7
45–54 years	357,981 (16.9)	45,678 (11.6)	48.6	38.7	12.7
55–64 years	321,265 (15.1)	30,986 (7.9)	49.0	37.3	13.7
65–74 years	249,543 (11.8)	25,602 (6.5)	48.1	37.7	14.2
≥ 75 years	182,341 (8.6)	54,667 (13.9)	39.5	38.9	21.6
Sex					
Male	1,053,185 (49.6)	181,250 (46.2)	45.7	42.8	11.5
Female	1,069,006 (50.4)	210,934 (53.8)	51.0	37.2	11.8
Intersex or indeterminate	–	17 (< 0.1)	76.5	23.5	0
Missing	–	37 (< 0.1)	97.3	2.7	0
Aboriginal and/or Torres Strait Islander status					
Yes	88,693 (3.2)	29,785 (7.6)	42.8	48.1	9.1
No	2,661,171 (96.8)	351,635 (89.6)	48.5	39.3	12.2
Missing	–	10,818 (2.8)	67.1	32.9	0
Residential remoteness					
Major cities	1,747,292 (78.7)	237,340 (60.5)	48.6	39.3	12.2
Inner regional	194,267 (8.8)	61,484 (15.7)	48.1	39.2	12.6
Outer regional	156,306 (7.0)	32,256 (8.2)	49.6	39.0	11.4
Remote	69,469 (3.1)	20,304 (5.2)	51.6	38.3	10.1
Very remote	52,440 (2.4)	22,102 (5.6)	45.4	44.2	10.4
Missing	–	18,752 (4.8)	48.5	46.1	5.4
Socioeconomic status					
Quintile 1 (Most disadvantaged)	50,366 (1.9)	16,380 (4.2)	42.8	47.0	10.3
Quintile 2	220,557 (8.3)	18,341 (4.7)	51.3	37.3	11.5
Quintile 3	562,468 (21.2)	53,724 (13.7)	50.8	38.0	11.3
Quintile 4	741,194 (27.9)	122,253 (31.2)	44.9	45.9	9.2
Quintile 5 (Least disadvantaged)	1,079,150 (40.7)	162,788 (41.5)	50.9	34.6	14.5
Missing	–	18,752 (4.8)	48.5	46.1	5.4

^a^Western Australian population aged ≥ 18 years for age and sex categories; aged ≥ 0 years for Aboriginal and/or Torres Strait Islander status and socioeconomic status categories; aged ≥ 15 years for residential remoteness categories; (Australian Bureau of Statistics, 2022; Hodges et al., 2020; Hoek, 2006)

Within care settings, 282,321 individuals (72.0%) had at least one contact with community mental health service, 223,755 individuals (57.0%) had at least one contact with ED mental health service, and 152,820 (39.0%) had at least one contact with an inpatient mental health service. Patient characteristics at first contact within the collection period in each care setting are presented in Table S4. Of note, a higher proportion of individuals had their first inpatient mental health service contact at age ≥ 75 years compared to individuals who had their first ED or community mental health service contact at age ≥ 75 years (17.3% for inpatient; 12.8% for ED; 10.8% for community). Similarly, higher proportion of individuals had their first inpatient contact when they were residing in the least disadvantaged areas compared to individuals who had their first ED or community contact when they were residing in the least disadvantaged areas (49.2% for inpatient; 43.4% for community; 36.0% for ED). In contrast, 4.8% of those who had an ED contact had their first contact when they were residing in the most disadvantaged areas compared to 3.9%, respectively, for those who had a community or inpatient contact.

#### First Recorded Relevant Mental Health Conditions

Of the 22 mental health conditions, the eight conditions with the highest number of individuals with at least one service contact were major depressive disorders (70,835; 18.1%), reaction to severe stress and adjustment disorders (70,044; 17.9%), anxiety disorders (63,658; 16.2%), alcohol use disorder (51,641; 13.2%), organic mental disorders not including dementia (37,147; 9.5%), drug use disorders (31,092; 7.9%), dementia (22,174; 5.7%), and schizotypal and delusional disorders (21,228; 5.4%) (Table 2). The percentages of individuals at first recorded relevant conditions were generally highest in the younger age groups (age 18–34 years) and decreased with age except for dementia and organic mental disorders not including dementia, which 80.2% and 54.2%, respectively, of individuals at first record were in the aged ≥ 75 years group (Table 2 and Table S5). Compared with males, lower percentages of females had a record of alcohol use disorder (58.4% vs 41.6%), drug use disorder (63.2% vs 36.8%), schizotypal and delusional disorders (57.8% vs 42.2%), schizophrenia and schizoaffective disorders (62.0% vs 38.0%), early onset behavioural and emotional disorders (58.0% vs 42.0%), and disorder of psychological development (72.2% vs 27.7%). In contrast, higher percentages of females had a record of major depressive disorders (41.8% vs 58.2%), reaction to severe stress and adjustment disorders (44.4% vs 55.6%), anxiety disorders (37.0% vs 62.9%), specific personality disorders (33.7% vs 66.3%), bipolar disorders (40.4% vs 59.6%), behavioural syndromes associated with physiological disturbances and physical factors (9.9% vs 90.1%), somatoform disorders (33.8% vs 66.2%), dissociative disorders (28.8% vs 71.2%), obsessive compulsive disorders (41.4% vs 58.6%), other affective disorders (41.3% vs 58.7%), and other neurotic disorders (37.8% vs 62.2%). Sex-specific patient characteristics are not reported due to the small number of individuals in some subgroups.

**Table 2:** Characteristics of individuals on their first recorded relevant mental health conditions^a^ over age 18 years since 2005

Mental health conditions^a^ (ICD-10-AM)	Major depressive disorders (F32-F33, F34.1)	Reaction to severe stress, and adjustment disorders (F43)	Anxiety disorders (F40-F41)	Alcohol use disorder (F10)	Organic mental disorders not including dementia (F04-F09)	Drug use disorders (F11-F19)	Dementia (F00-F03)	Schizotypal and delusional disorders (F21-F24, F28-F29)
All	70,835	70,044	63,658	51,641	37,147	31,092	22,174	21,228
Age (%)								
18–24 years	19.7	24.5	20.8	29.1	3.6	27.6	< 0.1	23.1
25–34 years	21.8	25.6	23.5	22.6	5.2	33.8	0.1	27.7
35–44 years	19.6	20.8	18.3	19.0	5.7	22.0	0.2	20.3
45–54 years	15.7	14.4	13.4	14.6	6.8	10.4	0.9	13.8
55–64 years	9.4	7.4	9.3	8.0	9.3	3.4	3.9	6.5
65–74 years	6.2	3.5	6.8	4.3	15.2	1.5	14.7	4.1
≥ 75 years	7.7	3.9	7.8	2.4	54.2	1.3	80.2	4.6
Sex (%)								
Male	41.8	44.4	37.0	58.4	49.2	63.2	46.5	57.8
Female	58.2	55.6	62.9	41.6	50.8	36.8	53.5	42.2
Residential remoteness (%)								
Major cities	63.6	62.5	62.7	62.3	74.3	62.9	72.3	65.8
Inner regional	16.7	17.1	16.3	13.2	13.0	15.1	15.2	14.8
Outer regional	7.7	7.3	9.2	6.4	6.0	8.3	7.2	6.2
Remote	4.9	4.6	5.0	4.5	2.4	4.2	3.0	3.2
Very remote	4.4	4.5	3.8	7.0	1.9	3.8	1.2	3.9
Missing	2.6	4.0	3.0	6.7	2.4	5.6	1.2	6.0
Socioeconomic status (%)								
Quintile 1	3.0	3.4	2.7	5.7	1.7	3.2	1.5	3.1
Quintile 2	4.4	5.0	5.0	4.3	3.7	5.5	3.8	4.6
Quintile 3	13.6	13.9	14.4	11.4	12.9	14.3	14.3	13.2
Quintile 4	31.4	35.0	34.3	31.9	31.2	32.1	28.4	30.8
Quintile 5	45.0	38.7	40.6	40.0	48.2	39.3	50.8	42.2
Missing	2.6	4.0	3.0	6.7	2.4	5.6	1.2	6.0
Mental health care setting (%)								
Community	35.0	16.6	18.5	1.9	63.5	8.1	17.7	21.6
ED	30.3	55.2	63.5	69.8	7.0	48.7	31.4	52.9
Inpatient	34.7	28.2	17.9	28.3	29.5	43.2	50.9	25.5

^a^The eight mental health conditions with the highest number of individuals are included in this Table. Refer to Table S6 for other mental health conditions;

ICD-10-AM = the International Statistical Classification of Diseases and Related Health Problems, Tenth Revision, Australian Modification; ED = emergency department

Nine mental health conditions had > 50% of first records in the ED setting. These were other neurotic disorders (90.7%), early onset behavioural and emotional disorders (75.7%), alcohol use disorder (69.8%), unspecified mental disorder (63.9%), anxiety disorders (63.5%), other personality and behavioural disorders (55.8%), reaction to severe stress, and adjustment disorders (55.2%), somatoform disorders (54.4%), and schizotypal and delusional disorders (52.9%). Of these, four were in the top eight conditions with the highest number of individuals with at least one contact. In comparison, only four conditions in community setting (one in top eight) and two in inpatient setting (one in top eight) had > 50% of first records (Table 2 and Table S5). Mental health care setting on first recorded conditions by patient characteristics are presented in Table S6.

### Access of Mental Health Service

**Fig. 1:**
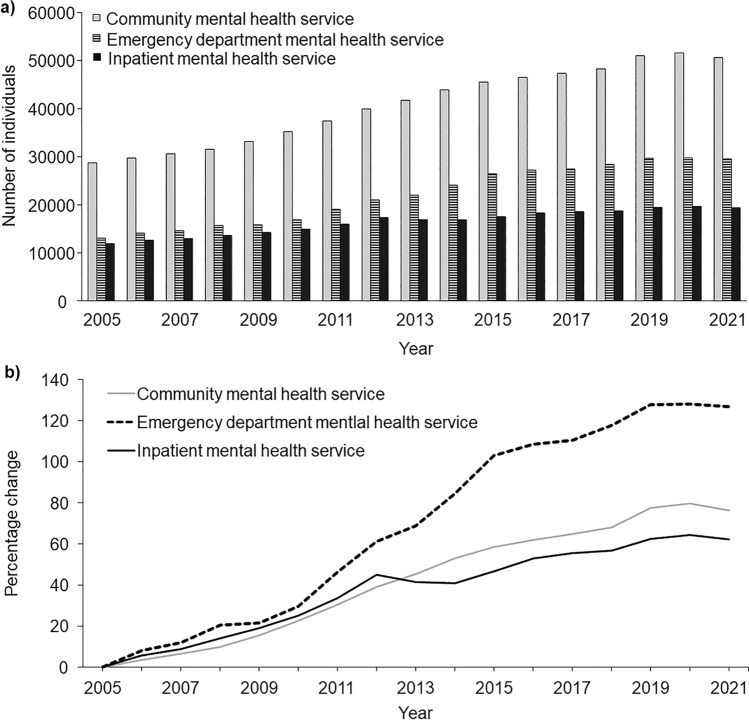
**a** Number of individuals, and **b** change in number of individuals relative to 2005, who accessed state-funded mental health services by care setting and year

The number of individuals who accessed community mental health services each year was substantially higher than for EDs and inpatient mental health services (e.g., values in 2021 were 50,690 for community; 29,524 for ED; 19,403 for inpatient), but the percentage increase over time was substantially higher for mental health services accessed through EDs than for community or inpatient mental health services (e.g., values in 2021 were 76.2% for community; 126.6% for ED; 62.5% for inpatient; Fig. 1). When stratified by sex, the percentage change increased linearly for females but seemingly decreased during the first two years of the COVID-19 pandemic for males (Fig S1). For inpatient mental health service, when a stricter definition was applied, 9239 individuals were no longer considered to have had at least one contact. Of note, the difference between the percentage change in the number of individuals relative to 2005 derived from the stricter definition and the definition applied throughout this study increased from around 2015 (e.g., 0.9% in 2006, 5.2% in 2015, 10.7% in 2021; Fig S2).

The number of individuals over time for the ten mental health conditions with the highest number of individuals with at least one contact within a care setting are presented in Fig S3–S5. Of note, for reaction to severe stress and adjustment disorders, the number of individuals with at least one ED contact increased between 2005 and 2021 (from 1540 in 2005 to 5395 in 2021). While the number of individuals with at least one inpatient contact also increased between 2005 and 2013, there was a drop in numbers between 2013 and 2017 (from 1655 in 2005 to 2937 in 2013 and 1914 in 2017). During this period, the number of individuals with at least one community contact increased from being relatively stable before 2014. In contrast, for major depressive disorders, the number of individuals with at least one community contact decreased between 2005 and 2021 (from 4822 in 2005 to 2650 in 2021), while the number of individuals with at least one inpatient contact increased over the same period (from 2564 in 2005 to 3163 in 2021) and the number of individuals with at least one ED contact increased between 2005 and 2015, and by 2021 decreased to numbers close to those in 2009–2010 (from 1293 in 2005 to 2441 in 2015 and 1618 in 2021).

### Pattern of Access

**Fig. 2:**
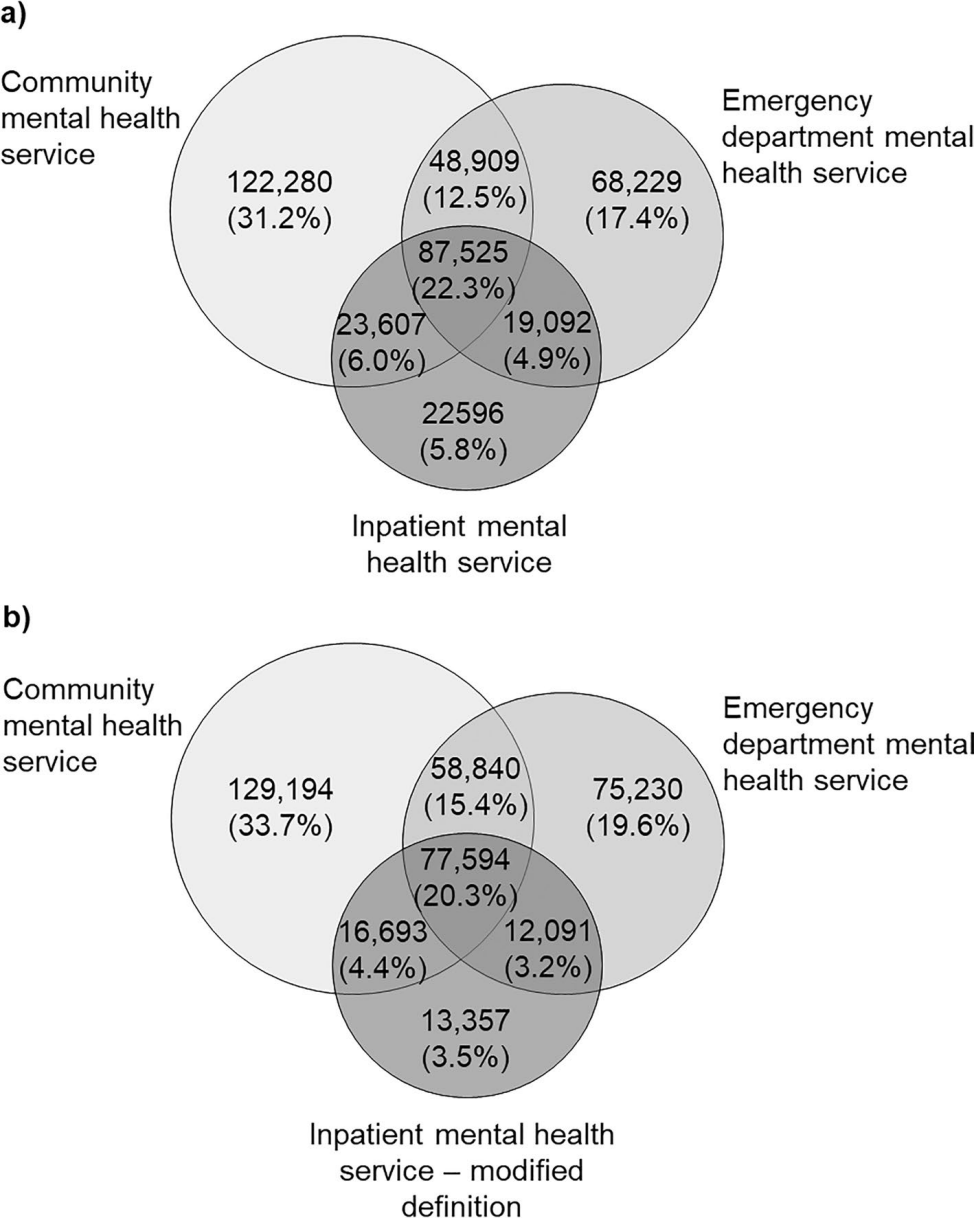
Pattern of access of state-funded mental health services for the period 2005–2021 **a** using original definition of inpatient mental health service, and **b** using a modified (stricter) definition of inpatient mental health service

**Fig. 3:**
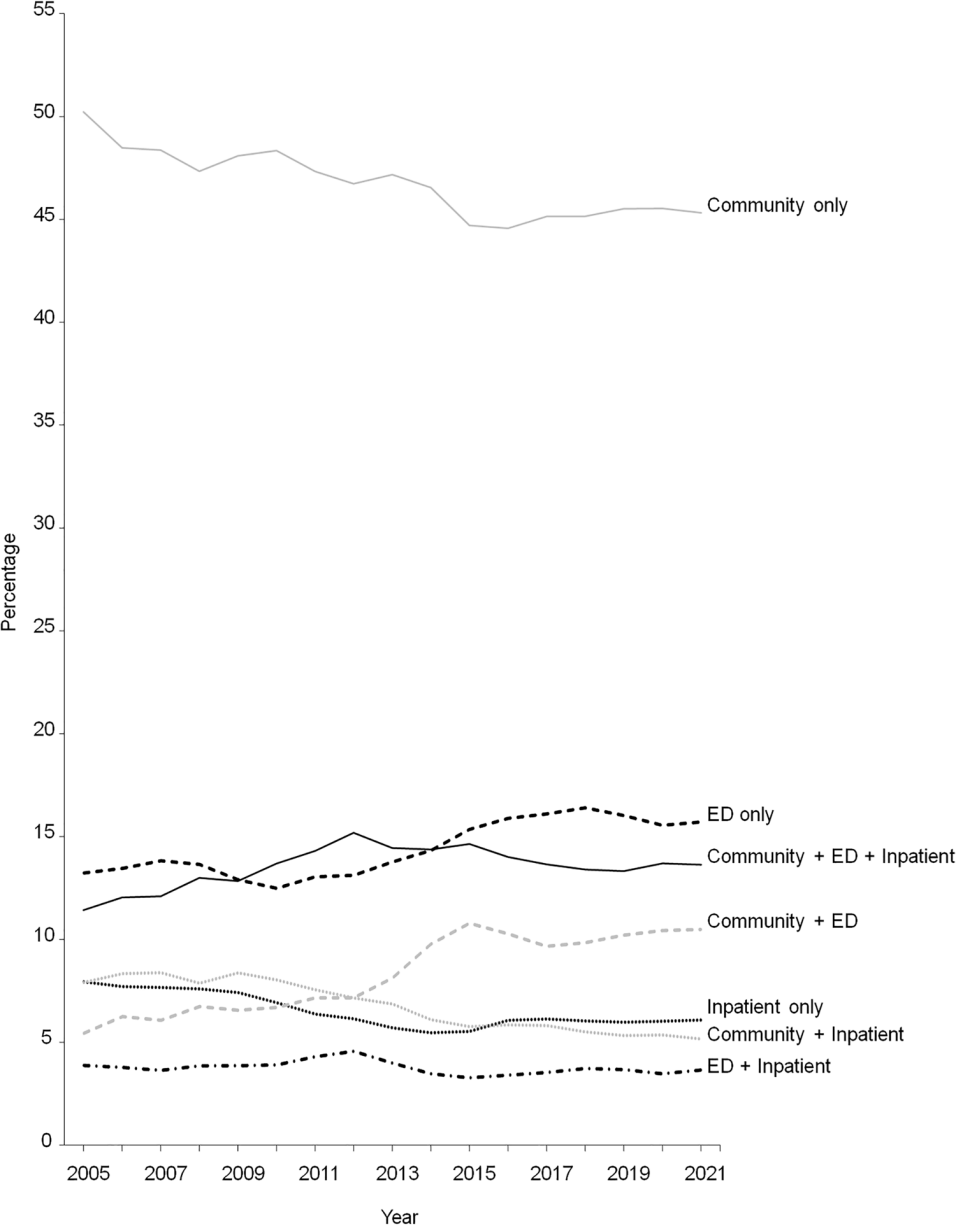
Pattern of access of state-funded mental health services (community, emergency department (ED), inpatient) by year

For the period 2005–2021, 22.3% of the cohort accessed mental health services from all three care settings, 23.4% accessed from two care settings and 54.3% from only one care setting (Fig. 2a). While a community mental health service was the most common setting accessed by individuals who accessed a mental health service from only one care setting (31.2%), the community + ED combination was the most common setting accessed by those who accessed service from two care settings. When studying the pattern of access over time, the percentages declined for community only, inpatient only, community + inpatient combination and increased for ED only and community + ED combination (Fig. 3). Similar patterns were observed between males and females (Fig S6–S7). When the stricter inpatient mental health service definition was applied, the percentages of the care setting combinations accessed that did not include inpatient increased, but the ranking of the combination remained unchanged (Fig. 2b).

## Discussion

Using a contemporary dataset with long follow-up and population-wide coverage, our study showed that in the Western Australian adult population, females, people with an Aboriginal and/or Torres Strait Islander background, and those who lived outside major cities or in the most disadvantaged areas were more likely to access state-funded mental health services. While the first mental health service contact commonly occurred in the community setting, differences were observed between population subgroups, care settings, and mental health conditions. Furthermore, changes were observed in the pattern of access to mental health service over time.

These findings are consistent with the Australian mental health system (AIHW, 2023a), whereby individuals with greater personal resources usually visit a general practitioner for initial assessment and then are either managed by the general practitioner or referred to a specialist. These services are provided by the private health system and are not captured in our dataset. Thus, individuals included in our study were likely to have mental health conditions that required ED and/or in-hospital treatments, or from lower socioeconomic backgrounds and were unable to afford private specialist services. Moreover, with the vast geographical area and very low population density outside the major city, there are limited options for specialist services in regional and remote WA (Kaleveld et al., 2023).

With respect to age, a similar pattern was observed between the care settings in which the percentages of individuals at first contact were highest in the youngest age groups, decreased with age, and followed by an increase in the group ≥ 75 years. This is consistent with the age at onset of most mental health conditions occurring by young adulthood (Solmi et al., 2022). The proportion of individuals who had their first inpatient contact at age ≥ 75 years was also higher than that for first community or ED contacts at age ≥ 75 years. This is due to dementia, which has a disease onset much later in life than other mental health conditions. Nationally, 62% of individuals with an inpatient stay due to dementia in 2020–21 were aged 75–89 years (AIHW, 2023b).

Unsurprisingly, sex differences were observed by mental health conditions, where alcohol and drug use disorders were more prevalent in males and affective disorders were more prevalent in females (McHugh et al., 2018; Swaab & Bao, 2020). Moreover, 90% of the cohort with recorded behavioural syndromes and associated with physiological disturbances and physical factors were females as individuals in this category were mainly recorded with eating disorders and mental and behavioural disorders associated with the puerperium, not elsewhere classified, which are conditions predominantly affecting females (Hoek, 2006).

The number of individuals accessing state-funded mental health service each year have increased steadily since 2005 in all three care settings. The increase has been particularly pronounced for services accessed through EDs, where the number of individuals with at least one contact in a year doubled in just over a decade, which is consistent with national trends reported for similar period (Tran et al., 2020). The main mental health conditions contributing to this increase were alcohol disorder, reaction to severe stress and adjustment disorders, and anxiety disorders. The increase in ED presentations for alcohol disorder contradicts with the decrease in the prevalence of high-risk alcohol consumption in the Western Australian general population over similar period (Epidemiology Directorate, 2021). Nevertheless, risky drinking is more likely to occur in people with mental health conditions than those without the conditions (AIHW, 2020). The increase in acute severe stress reaction is possibly masking people with another underlying mental health condition who are presenting to ED in a situational crisis. For example, specific personality disorder was not included in the ten mental health conditions with the most individuals with an ED mental health service contact despite people with the disorder are likely to present at EDs and re-present within 28 days (Collins et al., 2020; Lewis et al., 2019). As ED is not an ideal care setting for managing mental health conditions, education programs on where the public can seek support for acute and chronic symptoms of mental health challenges outside ED will be required to divert care away from EDs to more appropriate care settings i.e. community mental health service or private specialist service.

The pattern of access has also changed. While over half of the cohort only accessed services from one care setting during the 17-year period, over one-fifth accessed services from all three settings. The percentages of individuals accessing service from EDs only and both community and EDs have increased. These changes suggested increases in either patient needs, mental health service resources, or both, over time.

The widening gap since 2015 in our results that compared two definitions of inpatient mental health services has potential implications on costing and resource allocation. While the stricter definition identified individuals with a clear mental health admission, the widening gap suggests there are increasingly more individuals with mental and behavioural disorders that have led to an admission.

The major strength of our study was the use of linked health administrative records, which provided the usual advantages of administrative health data resources including population coverage, long-term follow-up, and very little missing data in crucial fields of patient information. Nevertheless, our linked dataset also included the usual limitations of health administrative records (Mazzali & Duca, 2015). Furthermore, we do not have information on mental health services provided by private specialists, primary care providers, non-government managed organisations nor prescriptions of psychotropics. Our dataset also lacked records on mental health service contacts that occurred before aged 18 years. The descriptive nature of our analysis meant that patterns over time or patient characteristics associated with accessing mental health services may not be causally related.

We have outlined some interesting issues on the use of state-funded mental health services in WA through describing the state-funded mental health service adult user population and their service access pattern over a 17-year period. Further research on, for example, examining pattern of access and patient pathways through the care system for specific mental health disorders and whether the pathways differ by patient characteristics will be required to support individuals with mental health conditions by improving access to mental health care.

## Supplementary Information

Below is the link to the electronic supplementary material.

Supplementary file1 (DOCX 1200 KB)

## Data Availability

The data that support the findings of this study are available from the Government of Western Australia Department of Health (https://www.datalinkage-wa.org.au/). Restrictions apply to the availability of these data, which were used under license for the current study, and so are not publicly available.
