# Correction: Patterns of Mental Health Service Utilisation: A population-Based Linkage of Over 17 Years of Health Administrative Records

**DOI:** 10.1007/s10597-025-01464-x

**Published:** 2025-04-03

**Authors:** Crystal Man Ying Lee, Kevin Chai, Peter M. McEvoy, Kyran Graham-Schmidt, Daniel Rock, Kim S. Betts, Justin Manuel, Mathew Coleman, Shiv Meka, Rosa Alati, Suzanne Robinson

**Affiliations:** 1 School of Population Health, Curtin University, Perth, WA Australia; 2 Centre for Clinical Interventions, North Metropolitan Health Service, Perth, WA Australia; 3 Department of Health, Perth, WA Australia; 4 WA Primary Health Alliance, Perth, WA Australia; 5 Discipline of Psychiatry, Medical School, University of Western Australia, Perth, WA Australia; 6 Faculty of Health, University of Canberra, Canberra, ACT Australia; 7 WA Country Health Service, Perth, WA Australia; 8 WA Country Health Service, Albany, WA Australia; 9 East Metropolitan Health Service, Perth, WA Australia; 10 Deakin Health Economics, Deakin University, Melbourne, VIC Australia

**Correction to: Community Mental Health Journal (2024) 60:1472–1483**

10.1007/s10597-024-01300-8

The original version of this article unfortunately contained an error in text.

In first paragraph of “First Recorded Relevant Mental Health Conditions” section, the sentences beginning ‘Compared with males…’ and ‘In contrast,…”, were published incorrectly with the term ‘higher’ and ‘lower’ switched. Therefore, both incorrect and corrected text is given here.

**Incorrect text**:

Compared with males, higher percentages of females had a record of alcohol use disorder (58.4% vs. 41.6%), drug use disorder (63.2% vs. 36.8%), schizotypal and delusional disorders (57.8% vs. 42.2%), schizophrenia and schizoaffective disorders (62.0% vs. 38.0%), early onset behavioural and emotional disorders (58.0% vs. 42.0%), and disorder of psychological development (72.2% vs. 27.7%). In contrast, lower percentages of females had a record of major depressive disorders (41.8% vs. 58.2%), reaction to severe stress and adjustment disorders (44.4% vs. 55.6%), anxiety disorders (37.0% vs. 62.9%), specific personality disorders (33.7% vs. 66.3%), bipolar disorders (40.4% vs. 59.6%), behavioural syndromes associated with physiological disturbances and physical factors (9.9% vs. 90.1%), somatoform disorders (33.8% vs. 66.2%), dissociative disorders (28.8% vs. 71.2%), obsessive compulsive disorders (41.4% vs. 58.6%), other affective disorders (41.3% vs. 58.7%), and other neurotic disorders (37.8% vs. 62.2%).

**The correct text should read as**:

Compared with males, lower percentages of females had a record of alcohol use disorder (58.4% vs. 41.6%), drug use disorder (63.2% vs. 36.8%), schizotypal and delusional disorders (57.8% vs. 42.2%), schizophrenia and schizoaffective disorders (62.0% vs. 38.0%), early onset behavioural and emotional disorders (58.0% vs. 42.0%), and disorder of psychological development (72.2% vs. 27.7%). In contrast, higher percentages of females had a record of major depressive disorders (41.8% vs. 58.2%), reaction to severe stress and adjustment disorders (44.4% vs. 55.6%), anxiety disorders (37.0% vs. 62.9%), specific personality disorders (33.7% vs. 66.3%), bipolar disorders (40.4% vs. 59.6%), behavioural syndromes associated with physiological disturbances and physical factors (9.9% vs. 90.1%), somatoform disorders (33.8% vs. 66.2%), dissociative disorders (28.8% vs. 71.2%), obsessive compulsive disorders (41.4% vs. 58.6%), other affective disorders (41.3% vs. 58.7%), and other neurotic disorders (37.8% vs. 62.2%).

The original article has been corrected.

