# Supplementary material for: Patterns of Mental Health Service Utilisation: A Population-Based Linkage of Over 17 Years of Health Administrative Records
Source: Community Ment Health J. 2024 Jun 12;60(8):1472–83. doi: 10.1007/s10597-024-01300-8 (PMC11579069; doi:10.1007/s10597-024-01300-8)
Supplement: Supplementary file 1 — Supplementary file1 (DOCX 1200 KB) [file 10597_2024_1300_MOESM1_ESM.docx]

**Patterns of mental health service utilisation: a population-based linkage of over 17 years of health administrative records**

Journal name: *Community Mental Health Journal*

Crystal Man Ying Lee, Kevin Chai, Peter M McEvoy, Kyran Graham-Schmidt, Daniel Rock, Kim S Betts, Justin Manuel, Mathew Coleman, Shiv Meka, Rosa Alati, Suzanne Robinson

Correspondence to: Crystal Lee at Curtin University (crystal.lee@curtin.edu.au)

**Table S1** Description of the International Statistical Classification of Diseases and Related Health Problems, Tenth Revision, Australian Modification (ICD-10-AM) codes used to identify the project cohort

| **ICD-10-AM** | **Description** |
| --- | --- |
| F00-F99 | Mental and behavioural disorders |
| O99.3 | Mental disorders and diseases of the nervous system in pregnancy, childbirth and the puerperium |
| Q86.0 | Fetal alcohol syndrome (dysmorphic) |
| R44 | Other symptoms and signs involving general sensations and perceptions |
| R45 | Symptoms and signs involving emotional state |
| R46 | Symptoms and signs involving appearance and behaviour |
| T39-50 | Poisoning by drugs, medicaments and biological substances |
| T51-52 | Toxic effect of alcohol and organic solvents |
| T58 | Toxic effect of carbon monoxide |
| X60-84 | Intentional self-harm |
| Y87.0 | Sequelae of intentional self-harm |
| Z00.4 | General psychiatric examination, not elsewhere classified |
| Z03.2 | Observation for suspected mental and behavioural disorders |
| Z04.6 | General psychiatric examination, requested by authority |
| Z09.3 | Follow-up examination after psychotherapy |
| Z13.3 | Special screening examination for mental and behavioural disorders |
| Z59-65 | Persons with potential health hazards related to socioeconomic or psychosocial circumstances |
| Z71.4-71.5 | Counselling and surveillance for alcohol or drug use disorder |
| Z72-73 | Problems related to lifestyle and life-management difficulty |
| Z86.4-86.5 | Personal history of psychoactive substance use disorder or other mental and behavioural disorders |
| Z91.5-91.6 | Personal history of self-harm or other physical trauma |
| U79 | Mental and behavioural disorders |

**Table S2** Mental health conditions by the International Statistical Classification of Diseases and Related Health Problems, Tenth Revision, Australian Modification (ICD-10-AM) codes

| **ICD-10-AM** | **Mental health condition** |
| --- | --- |
| F00-F03 | Dementia |
| F04-F09 | Organic mental disorders not including dementia |
| F10 | Alcohol use disorder |
| F11-F19 | Drug use disorder |
| F20, F25 | Schizophrenia and schizoaffective disorders |
| F21-F24, F28-F29 | Schizotypal and delusional disorders |
| F30-F31, F34.0 | Bipolar disorders |
| F32-F33, F34.1 | Major depressive disorders |
| F34 (excluding 34.0 and 34.1), F38-F39 | Other affective disorders |
| F40-F41 | Anxiety disorders |
| F42 | Obsessive compulsive disorders |
| F43 | Reaction to severe stress and adjustment disorders |
| F44 | Dissociative disorders |
| F45 | Somatoform disorders |
| F48 | Other neurotic disorders |
| F50-F59 | Behavioural syndromes associated with physiological disturbances and physical factors |
| F60 | Specific personality disorders |
| F61-F69 | Other personality and behavioural disorder |
| F70-F79 | Mental retardation |
| F80-F89 | Disorders of psychological development |
| F90-F98 | Early onset behavioural and emotional disorders |
| F99 | Unspecified mental disorder |

**Table S3** Characteristics of individuals on their first contact with the Western Australian state funded mental health service over age 18 years since 2005 by sex

|  | **Male** | | | | **Female** | | | |
| --- | --- | --- | --- | --- | --- | --- | --- | --- |
|  | **N (%)** | **Mental health care setting at first contact (%)** | | | **N (%)** | **Mental health care setting at first contact (%)** | | |
|  |  | **C** | **ED** | **I** |  | **C** | **ED** | **I** |
| **All (age≥18 years)** | 18,1250 | 45.7 | 42.8 | 11.5 | 210,934 | 51.0 | 37.2 | 11.8 |
| Age |  |  |  |  |  |  |  |  |
| 18-24 years | 39,747 (21.9) | 44.3 | 48.2 | 7.5 | 49,517 (23.5) | 49.5 | 42.6 | 7.9 |
| 25-34 years | 37,230 (20.5) | 45.6 | 45.8 | 8.6 | 45,780 (21.7) | 60.5 | 32.2 | 7.3 |
| 35-44 years | 29,975 (16.5) | 47.6 | 41.6 | 10.8 | 33,009 (15.6) | 54.8 | 34.7 | 10.6 |
| 45-54 years | 22,555 (12.4) | 49.2 | 39.3 | 11.6 | 23,117 (11.0) | 48.1 | 38.1 | 13.8 |
| 55-64 years | 15,463 (8.5) | 50.0 | 36.9 | 13.1 | 15,523 (7.4) | 48.0 | 37.6 | 14.4 |
| 65-74 years | 12,897 (7.1) | 47.4 | 38.4 | 14.2 | 12,705 (6.0) | 48.8 | 37.0 | 14.3 |
| ≥75 years | 23,383 (12.9) | 38.7 | 39.9 | 21.4 | 31,283 (14.8) | 40.1 | 38.1 | 21.8 |
| Aboriginal and/or Torres Strait Islander status | | | | | | | | |
| Yes | 14,451 (8.0) | 41.4 | 48.8 | 9.8 | 15,333 (7.3) | 44.1 | 47.4 | 8.5 |
| No | 161,502 (89.1) | 45.4 | 42.5 | 12.1 | 190,104 (90.1) | 51.1 | 36.6 | 12.4 |
| Missing | 5297 (2.9) | 65.3 | 34.7 | 0 | 5497 (2.6) | 68.6 | 31.4 | 0 |
| Residential remoteness |  |  |  |  |  |  |  |  |
| Major cities | 106,944 (59.0) | 45.0 | 43.0 | 12.0 | 130,375 (61.8) | 51.5 | 36.2 | 12.3 |
| Inner regional | 28,210 (15.6) | 46.1 | 41.1 | 12.9 | 33,269 (15.8) | 49.9 | 37.7 | 12.4 |
| Outer regional | 15,322 (8.5) | 48.8 | 40.0 | 11.3 | 16,930 (8.0) | 50.4 | 38.0 | 11.6 |
| Remote | 9875 (5.4) | 51.8 | 38.6 | 9.5 | 10,427 (4.9) | 51.4 | 37.9 | 10.7 |
| Very remote | 10,752 (5.9) | 44.0 | 45.3 | 10.7 | 11,347 (5.4) | 46.8 | 43.2 | 10.0 |
| Missing | 10,147 (5.6) | 43.3 | 50.8 | 5.9 | 8586 (4.1) | 54.6 | 40.5 | 4.9 |
| Socioeconomic status | | | | | | | | |
| Quintile 1 (Most disadvantaged) | 8113 (4.5) | 42.3 | 47.3 | 10.4 | 8266 (3.9) | 43.2 | 46.6 | 10.1 |
| Quintile 2 | 8949 (4.9) | 51.4 | 37.1 | 11.5 | 9390 (4.5) | 51.2 | 37.4 | 11.4 |
| Quintile 3 | 25,425 (14.0) | 48.9 | 39.7 | 11.4 | 28,295 (13.4) | 52.4 | 36.5 | 11.1 |
| Quintile 4 | 56,678 (31.3) | 41.7 | 48.8 | 9.4 | 65,567 (31.1) | 47.6 | 43.4 | 9.0 |
| Quintile 5 (Least disadvantaged) | 71,938 (39.7) | 47.7 | 38.2 | 14.2 | 90,830 (43.1) | 53.4 | 31.8 | 14.8 |
| Missing | 10,147 (5.6) | 43.3 | 50.8 | 5.9 | 8586 (4.1) | 54.6 | 40.5 | 4.9 |

C = community; ED = emergency department; I = inpatient;

**Table S4** Characteristics of individuals on their first contact with the respective mental health care settings over age 18 years since 2005 by sex

|  | **All** | | | **Male** | | | **Female** | | | |
| --- | --- | --- | --- | --- | --- | --- | --- | --- | --- | --- |
|  | **C^a^**  **%** | **ED^a^**  **%** | **I^a^**  **%** | **C^a^**  **%** | **ED^a^**  **%** | **I^a^**  **%** | **C^a^**  **%** | **ED^a^**  **%** | **I^a^**  **%** | |
| **N** | 282,321 | 223,755 | 152,820 | 128,286 | 109,769 | 74,173 | 153,985 | 113,976 | 78,644 | |
| Age |  |  |  |  |  |  |  |  |  | |
| 18-24 years | 22.8 | 24.2 | 19.2 | 21.7 | 23.2 | 18.4 | 23.7 | 25.1 | 19.9 | |
| 25-34 years | 23.4 | 21.1 | 19.4 | 21.9 | 22.5 | 20.5 | 24.8 | 19.7 | 18.3 | |
| 35-44 years | 17.3 | 16.4 | 16.8 | 17.7 | 17.1 | 17.5 | 16.9 | 15.7 | 16.1 | |
| 45-54 years | 12.0 | 11.9 | 12.8 | 13.3 | 11.9 | 12.8 | 11.0 | 11.9 | 12.8 | |
| 55-64 years | 7.7 | 7.6 | 8.0 | 8.5 | 7.7 | 8.3 | 7.0 | 7.5 | 7.7 | |
| 65-74 years | 6.0 | 6.0 | 6.5 | 6.6 | 6.2 | 6.9 | 5.5 | 5.8 | 6.2 | |
| ≥75 years | 10.8 | 12.8 | 17.3 | 10.2 | 11.5 | 15.5 | 11.3 | 14.2 | 18.9 | |
| Sex |  |  |  |  |  |  |  |  |  | |
| Male | 45.4 | 49.1 | 18.5 | - | - | - | - | - | - | |
| Female | 54.5 | 50.9 | 51.5 | - | - | - | - | - | - | |
| Aboriginal and/or Torres Strait Islander status | | | | | | | | | |  |
| Yes | 8.1 | 9.4 | 8.1 | 8.4 | 9.3 | 8.2 | 7.7 | 9.5 | 8.0 | |
| No | 88.8 | 88.5 | 91.9 | 88.2 | 88.5 | 91.8 | 89.4 | 88.5 | 92.0 | |
| Missing | 3.1 | 2.1 | 0 | 3.3 | 2.2 | 0 | 2.9 | 2.0 | 0 | |
| Residential remoteness | | | | | | | | | |  |
| Major cities | 60.5 | 60.8 | 63.9 | 58.6 | 60.3 | 62.7 | 62.0 | 61.2 | 64.9 | |
| Inner regional | 15.4 | 15.1 | 14.9 | 15.3 | 14.6 | 14.9 | 15.4 | 15.5 | 15.0 | |
| Outer regional | 8.4 | 8.3 | 8.3 | 8.9 | 8.1 | 8.4 | 8.0 | 8.4 | 8.3 | |
| Remote | 5.5 | 5.1 | 4.7 | 5.9 | 5.1 | 4.7 | 5.1 | 5.1 | 4.7 | |
| Very remote | 5.4 | 6.0 | 5.3 | 5.7 | 6.1 | 5.5 | 5.1 | 6.0 | 5.1 | |
| Missing | 4.9 | 4.8 | 2.9 | 5.5 | 5.8 | 3.9 | 4.4 | 3.7 | 2.0 | |
| Socioeconomic status | | | | | | | | | |  |
| Quintile 1 | 3.9 | 4.8 | 3.9 | 4.2 | 4.9 | 4.0 | 3.6 | 4.8 | 3.7 | |
| Quintile 2 | 5.1 | 4.6 | 4.8 | 5.5 | 4.6 | 5.0 | 4.7 | 4.7 | 4.7 | |
| Quintile 3 | 14.0 | 13.8 | 13.0 | 14.6 | 13.8 | 13.2 | 13.6 | 13.8 | 12.9 | |
| Quintile 4 | 28.7 | 36.0 | 26.1 | 28.6 | 35.6 | 26.3 | 28.8 | 36.4 | 26.0 | |
| Quintile 5 | 43.4 | 36.0 | 49.2 | 41.6 | 35.3 | 47.6 | 44.9 | 36.6 | 50.7 | |
| Missing | 4.9 | 4.8 | 2.9 | 5.5 | 5.8 | 3.9 | 4.4 | 3.7 | 2.0 | |

**^a^** Individuals included in the three care settings are not mutually exclusive;

C = community; ED = emergency department; I = inpatient;

**Table S5** Characteristics of individuals on their first recorded relevant mental health conditions over age 18 years since 2005

| **Mental health condition (ICD-10-AM)** | **Schizophrenia and schizoaffective disorders (F20, F25)** | **Specific personality disorders (F60)** | **Bipolar disorders (F30-F31, F34.0)** | **Unspecified mental disorder (F99)** | **Behavioural syndromes associated with physiological disturbances and physical factors (F50-F59)** | **Other personality and behavioural disorders (F61-F69)** | **Early onset behavioural and emotional disorders (F90-F98)** |
| --- | --- | --- | --- | --- | --- | --- | --- |
| **All** | 18,052 | 17,046 | 15,884 | 11,073 | 6745 | 4251 | 4245 |
| Age (%) |  |  |  |  |  |  |  |
| 18-24 years | 19.0 | 36.1 | 15.2 | 23.7 | 35.7 | 27.0 | 28.0 |
| 25-34 years | 28.3 | 27.8 | 23.9 | 24.3 | 37.8 | 26.2 | 23.1 |
| 35-44 years | 23.1 | 18.7 | 22.2 | 20.7 | 15.1 | 19.4 | 16.4 |
| 45-54 years | 14.9 | 11.0 | 18.2 | 15.2 | 3.8 | 12.3 | 10.1 |
| 55-64 years | 7.4 | 3.9 | 10.6 | 9.1 | 2.7 | 5.7 | 5.2 |
| 65-74 years | 4.4 | 1.5 | 6.0 | 3.6 | 2.2 | 4.0 | 4.8 |
| ≥75 years | 2.8 | 1.0 | 3.9 | 3.4 | 2.7 | 5.4 | 12.5 |
| Sex (%) |  |  |  |  |  |  |  |
| Male | 62.0 | 33.7 | 40.4 | 51.1 | 9.9 | 52.5 | 58.0 |
| Female | 38.0 | 66.3 | 59.6 | 48.9 | 90.1 | 47.4 | 42.0 |
| Residential remoteness (%) | | | | | | | |
| Major cities | 61.1 | 70.0 | 64.9 | 56.4 | 69.8 | 75.6 | 68.6 |
| Inner regional | 17.4 | 15.8 | 15.9 | 11.5 | 13.6 | 9.4 | 15.1 |
| Outer regional | 6.8 | 5.4 | 7.7 | 17.1 | 6.3 | 7.0 | 5.5 |
| Remote | 4.2 | 3.0 | 4.4 | 6.1 | 4.6 | 2.3 | 2.9 |
| Very remote | 5.4 | 2.3 | 3.5 | 5.0 | 3.8 | 1.5 | 2.5 |
| Missing | 5.1 | 3.6 | 3.5 | 3.8 | 1.9 | 4.3 | 5.5 |
| Socioeconomic status (%) | | | | | | | |
| Quintile 1 | 3.9 | 1.9 | 2.1 | 6.4 | 2.4 | 1.6 | 1.9 |
| Quintile 2 | 5.0 | 4.6 | 3.9 | 9.1 | 3.9 | 4.1 | 3.5 |
| Quintile 3 | 12.7 | 15.2 | 12.9 | 15.0 | 10.6 | 14.2 | 13.7 |
| Quintile 4 | 28.6 | 31.5 | 28.7 | 31.5 | 26.8 | 38.6 | 35.3 |
| Quintile 5 | 44.7 | 43.3 | 48.9 | 34.1 | 54.5 | 37.2 | 40.1 |
| Missing | 5.1 | 3.6 | 3.5 | 3.8 | 1.9 | 4.3 | 5.5 |
| Mental health care setting (%) | | | | | | | |
| Community | 49.0 | 44.4 | 39.2 | 35.2 | 50.1 | 20.8 | 12.9 |
| ED | 13.3 | 18.1 | 18.5 | 63.9 | 24.8 | 55.8 | 75.7 |
| Inpatient | 37.7 | 37.5 | 42.3 | 0.8 | 25.0 | 23.5 | 11.4 |

ICD-10-AM = the International Statistical Classification of Diseases and Related Health Problems, Tenth Revision, Australian Modification**;** ED= emergency department;

**Table S5** continued

| **Mental health condition (ICD-10-AM)** | **Somatoform disorders (F45)** | **Dissociative disorders (F44)** | **Obsessive compulsive disorders (F42)** | **Other affective disorders (F34 (excluding 34.0, 34.1), F38-F39)** | **Other neurotic disorders (F48)** | **Disorders of psychological development (F80-F89)** | **Mental retardation (F70-F79)** |
| --- | --- | --- | --- | --- | --- | --- | --- |
| **All** | 3003 | 2850 | 1917 | 1488 | 1136 | 837 | 591 |
| Age (%) |  |  |  |  |  |  |  |
| 18-24 years | 19.4 | 18.4 | 29.4 | 22.9 | 23.1 | 54.4 | 27.6 |
| 25-34 years | 20.2 | 22.4 | 30.9 | 24.1 | 24.3 | 24.4 | 19.6 |
| 35-44 years | 18.4 | 20.8 | 17.1 | 20.0 | 21.7 | 9.6 | 17.1 |
| 45-54 years | 16.5 | 17.5 | 10.2 | 15.5 | 13.5 | 6.2 | 15.6 |
| 55-64 years | 11.6 | 10.8 | 6.2 | 8.6 | 8.5 | 3.2 | 9.0 |
| 65-74 years | 7.2 | 5.6 | 3.6 | 4.4 | 3.7 | 1.7 | 8.6 |
| ≥75 years | 6.7 | 4.5 | 2.8 | 4.6 | 5.2 | 0.6 | 2.5 |
| Sex (%) |  |  |  |  |  |  |  |
| Male | 33.8 | 28.8 | 41.4 | 41.3 | 37.8 | 72.2 | 50.4 |
| Female | 66.2 | 71.2 | 58.6 | 58.7 | 62.2 | 27.7 | 49.6 |
| Residential remoteness (%) | | | | | | | |
| Major cities | 66.2 | 68.0 | 67.9 | 56.3 | 56.5 | 69.9 | 62.6 |
| Inner regional | 18.5 | 13.2 | 16.6 | 13.0 | 34.2 | 14.0 | 15.4 |
| Outer regional | 6.0 | 8.1 | 6.6 | 12.9 | 2.5 | 8.2 | 8.5 |
| Remote | 4.3 | 4.2 | 4.0 | 7.0 | 0.9 | 3.0 | 5.8 |
| Very remote | 2.3 | 3.5 | 3.2 | 8.6 | 1.2 | 3.0 | 3.2 |
| Missing | 2.6 | 3.0 | 1.7 | 2.2 | 4.7 | 1.9 | 4.6 |
| Socioeconomic status (%) | | | | | | | |
| Quintile 1 | 1.9 | 2.8 | 1.8 | 6.5 | 1.0 | 2.6 | 3.4 |
| Quintile 2 | 3.9 | 4.8 | 3.3 | 7.2 | 4.6 | 5.9 | 5.4 |
| Quintile 3 | 13.2 | 15.0 | 13.4 | 21.5 | 13.6 | 13.6 | 16.4 |
| Quintile 4 | 36.3 | 31.6 | 29.0 | 26.6 | 43.3 | 29.4 | 28.4 |
| Quintile 5 | 42.1 | 42.7 | 50.8 | 36.0 | 32.9 | 46.6 | 41.8 |
| Missing | 2.6 | 3.0 | 1.7 | 2.2 | 4.7 | 1.9 | 4.6 |
| Mental health care setting (%) | | | | | | | |
| Community | 9.4 | 4.0 | 64.0 | 40.9 | 3.2 | 56.5 | 50.4 |
| ED | 54.4 | 30.7 | 9.9 | 25.2 | 90.7 | 9.8 | 4.9 |
| Inpatient | 36.2 | 65.3 | 26.1 | 33.9 | 6.2 | 33.7 | 44.7 |

ICD-10-AM = the International Statistical Classification of Diseases and Related Health Problems, Tenth Revision, Australian Modification**;** ED= emergency department;

**Table S6** Characteristics of individuals on their first recorded relevant mental health conditions in the respective mental health care settings over age 18 years since 2005

| **Mental health condition (ICD-10-AM)** | **Mental health care setting** | **Major depressive disorders (F32-F33, F34.1)** | **Reaction to severe stress, and adjustment disorders (F43)** | **Anxiety disorders (F40-F41)** | **Alcohol use disorder (F10)** | **Organic mental disorders not including dementia (F04-F09)** | **Drug use disorders (F11-F19)** | **Dementia (F00-F03)** | **Schizotypal and delusional disorders (F21-F24, F28-F29)** |
| --- | --- | --- | --- | --- | --- | --- | --- | --- | --- |
| Age (%) |  |  |  |  |  |  |  |  |  |
| 18-24 years | n | 13,970 | 17,136 | 13,226 | 15,019 | 1348 | 8577 | <5 | 4894 |
|  | Community | 30.4 | 14.6 | 22.1 | 1.0 | 10.5 | 10.2 | - | 26.5 |
|  | ED | 38.4 | 61.5 | 64.7 | 83.8 | 75.5 | 46.3 | - | 49.7 |
|  | Inpatient | 31.1 | 23.9 | 13.3 | 15.2 | 14.0 | 43.5 | - | 23.8 |
| 25-34 years | n | 15,432 | 17,922 | 14,956 | 11,693 | 1938 | 10,498 | 12 | 5874 |
|  | Community | 33.4 | 15.7 | 19.7 | 1.7 | 10.2 | 7.4 | 33.3 | 19.2 |
|  | ED | 35.2 | 58.1 | 67.7 | 72.1 | 73.6 | 48.1 | 33.3 | 58.9 |
|  | Inpatient | 31.4 | 26.2 | 12.6 | 26.2 | 16.2 | 44.5 | 33.3 | 21.9 |
| 35-44 years | n | 13,906 | 14,552 | 11,666 | 9823 | 2125 | 6848 | 40 | 4300 |
|  | Community | 33.6 | 15.3 | 18.2 | 2.2 | 13.2 | 7.1 | 25.0 | 16.5 |
|  | ED | 30.5 | 53.2 | 64.6 | 61.2 | 70.4 | 49.9 | 12.5 | 59.7 |
|  | Inpatient | 35.9 | 31.4 | 17.1 | 36.6 | 16.5 | 43.0 | 62.5 | 23.8 |
| 45-54 years | n | 11,119 | 10,074 | 8533 | 7532 | 2525 | 3231 | 190 | 2938 |
|  | Community | 33.4 | 15.8 | 16.0 | 2.2 | 12.1 | 6.2 | 31.1 | 17.7 |
|  | ED | 28.4 | 51.1 | 64.0 | 60.8 | 71.0 | 53.0 | 23.7 | 55.8 |
|  | Inpatient | 38.2 | 33.1 | 20.1 | 37.1 | 16.9 | 40.9 | 45.3 | 26.5 |
| 55-64 years | n | 6647 | 5197 | 5949 | 4199 | 3442 | 1069 | 874 | 1381 |
|  | Community | 35.6 | 17.7 | 14.3 | 2.6 | 11.4 | 6.4 | 27.4 | 22.6 |
|  | ED | 25.2 | 48.2 | 63.0 | 59.6 | 70.7 | 55.3 | 27.5 | 48.1 |
|  | Inpatient | 39.3 | 34.0 | 22.7 | 37.8 | 18.0 | 38.4 | 45.2 | 29.3 |
| 65-74 years | n | 4395 | 2451 | 4359 | 2214 | 5633 | 476 | 3270 | 873 |
|  | Community | 46.0 | 26.7 | 17.0 | 3.4 | 7.9 | 10.3 | 23.1 | 30.1 |
|  | ED | 18.8 | 45.3 | 59.0 | 57.6 | 67.1 | 51.9 | 27.9 | 32.0 |
|  | Inpatient | 35.2 | 28.1 | 24.0 | 39.0 | 25.0 | 37.8 | 49.0 | 37.9 |
| ≥75 years | n | 5429 | 2712 | 4970 | 1242 | 20,136 | 393 | 17,787 | 968 |
|  | Community | 48.6 | 33.3 | 17.1 | 5.2 | 4.3 | 12.7 | 16.1 | 36.9 |
|  | ED | 15.1 | 44.3 | 49.2 | 56.4 | 57.8 | 39.2 | 32.3 | 19.5 |
|  | Inpatient | 36.3 | 22.5 | 33.7 | 38.5 | 38.0 | 48.1 | 51.6 | 43.6 |
| Sex (%) |  |  |  |  |  |  |  |  |  |
| Male | n | 29,604 | 31,090 | 23,583 | 30,157 | 18,286 | 19,646 | 10306 | 12,269 |
|  | Community | 31.1 | 13.1 | 16.5 | 2.0 | 7.4 | 8.5 | 16.9 | 21.6 |
|  | ED | 34.2 | 56.9 | 66.8 | 68.7 | 64.2 | 48.9 | 31.1 | 54.6 |
|  | Inpatient | 34.7 | 30.0 | 16.7 | 29.3 | 28.3 | 42.6 | 52.0 | 23.8 |
| Female | n | 41,230 | 38,949 | 40,072 | 21,483 | 18,860 | 11,445 | 11,868 | 8959 |
|  | Community | 37.8 | 19.4 | 19.7 | 1.7 | 6.7 | 7.3 | 18.5 | 21.6 |
|  | ED | 27.6 | 53.8 | 61.6 | 71.4 | 62.7 | 48.4 | 31.6 | 50.6 |
|  | Inpatient | 34.7 | 26.8 | 18.7 | 27.0 | 30.6 | 44.3 | 49.9 | 27.8 |
| Residential remoteness (%) | | | | | | | | | |
| Major cities | n | 45,075 | 43,777 | 39,888 | 32,149 | 27,612 | 19,566 | 16,025 | 13,968 |
|  | Community | 32.0 | 17.5 | 19.7 | 1.6 | 6.6 | 7.5 | 16.3 | 21.0 |
|  | ED | 35.8 | 56.2 | 63.8 | 76.3 | 61.7 | 50.0 | 34.5 | 56.9 |
|  | Inpatient | 32.3 | 26.4 | 16.5 | 22.1 | 31.6 | 42.6 | 49.2 | 22.0 |
| Inner regional | n | 11,843 | 11,988 | 10,395 | 6792 | 4830 | 4701 | 3362 | 3138 |
|  | Community | 37.6 | 15.1 | 17.0 | 2.1 | 9.4 | 10.7 | 22.3 | 27.4 |
|  | ED | 31.7 | 58.8 | 67.7 | 67.7 | 68.3 | 49.5 | 28.6 | 50.6 |
|  | Inpatient | 30.7 | 26.2 | 15.3 | 30.2 | 22.3 | 39.8 | 49.1 | 22.0 |
| Outer regional | n | 5452 | 5105 | 5866 | 3329 | 2227 | 2581 | 1594 | 1324 |
|  | Community | 38.2 | 15.2 | 13.4 | 10.9 | 6.7 | 6.7 | 13.6 | 20.0 |
|  | ED | 10.4 | 44.4 | 59.8 | 49.4 | 65.6 | 46.3 | 19.1 | 34.4 |
|  | Inpatient | 51.4 | 40.5 | 26.8 | 47.4 | 27.8 | 47.0 | 67.3 | 45.6 |
| Remote | n | 3450 | 3208 | 3202 | 2316 | 884 | 1309 | 672 | 684 |
|  | Community | 47.4 | 16.5 | 19.8 | 2.6 | 5.3 | 8.7 | 25.0 | 28.1 |
|  | ED | 4.0 | 46.7 | 53.5 | 47.1 | 67.1 | 39.3 | 7.7 | 22.7 |
|  | Inpatient | 48.6 | 36.9 | 26.7 | 50.3 | 27.6 | 52.0 | 67.3 | 49.3 |
| Very remote | n | 3151 | 3164 | 2412 | 3612 | 691 | 1184 | 257 | 836 |
|  | Community | 49.1 | 18.3 | 20.8 | 3.4 | 12.2 | 16.1 | 12.1 | 26.6 |
|  | ED | 2.3 | 40.3 | 51.1 | 34.7 | 63.0 | 25.8 | 15.6 | 17.1 |
|  | Inpatient | 48.6 | 41.4 | 28.1 | 62.0 | 24.9 | 58.1 | 72.4 | 56.3 |
| Missing | n | 1864 | 2802 | 1896 | 3444 | 903 | 1751 | 264 | 1278 |
|  | Community | 35.7 | 10.2 | 12.7 | 0.8 | 5.4 | 3.7 | 55.7 | 8.8 |
|  | ED | 45.1 | 70.6 | 78.9 | 84.6 | 82.5 | 58.4 | 27.7 | 72.9 |
|  | Inpatient | 19.2 | 19.2 | 8.4 | 14.6 | 12.1 | 37.9 | 16.7 | 18.3 |
| Socioeconomic status (%) | | | | | | | | | |
| Quintile 1 | n | 2118 | 2396 | 1744 | 2963 | 628 | 1008 | 334 | 663 |
|  | Community | 46.2 | 14.4 | 14.6 | 2.9 | 11.0 | 12.6 | 16.8 | 21.1 |
|  | ED | 3.4 | 46.5 | 58.2 | 38.3 | 64.2 | 38.5 | 14.1 | 22.8 |
|  | Inpatient | 50.4 | 39.2 | 27.2 | 58.9 | 24.8 | 48.9 | 69.2 | 56.1 |
| Quintile 2 | n | 3098 | 3482 | 3191 | 2227 | 1363 | 1698 | 851 | 986 |
|  | Community | 36.4 | 16.2 | 14.5 | 2.6 | 6.7 | 9.4 | 18.8 | 24.4 |
|  | ED | 16.0 | 51.6 | 59.8 | 51.5 | 65.5 | 49.2 | 21.6 | 39.7 |
|  | Inpatient | 47.6 | 32.2 | 25.7 | 45.9 | 27.8 | 41.4 | 59.6 | 35.9 |
| Quintile 3 | n | 9617 | 9760 | 9147 | 5907 | 4777 | 4438 | 3168 | 2805 |
|  | Community | 39.0 | 20.7 | 19.9 | 2.4 | 7.6 | 9.1 | 16.6 | 23.8 |
|  | ED | 27.0 | 50.2 | 62.7 | 65.0 | 60.8 | 49.8 | 29.0 | 50.3 |
|  | Inpatient | 34.0 | 29.1 | 17.4 | 32.7 | 31.6 | 41.2 | 54.4 | 25.9 |
| Quintile 4 | n | 22,251 | 24,514 | 21,819 | 16,468 | 11,585 | 9977 | 6289 | 6535 |
|  | Community | 32.3 | 14.0 | 15.2 | 1.7 | 6.7 | 7.1 | 16.5 | 19.4 |
|  | ED | 39.8 | 63.5 | 71.6 | 76.7 | 72.2 | 56.6 | 38.8 | 61.2 |
|  | Inpatient | 27.9 | 22.5 | 13.2 | 21.7 | 21.2 | 36.3 | 44.7 | 19.5 |
| Quintile 5 | n | 31,887 | 27,090 | 25,862 | 20,633 | 17891 | 12,220 | 11,268 | 8961 |
|  | Community | 34.7 | 18.3 | 22.0 | 1.9 | 7.1 | 8.6 | 17.7 | 24.1 |
|  | ED | 27.1 | 49.2 | 56.7 | 69.7 | 57.4 | 41.3 | 29.3 | 48.5 |
|  | Inpatient | 38.2 | 32.5 | 21.3 | 28.4 | 35.5 | 50.2 | 53.0 | 27.4 |
| Missing | n | 1864 | 2802 | 1896 | 3444 | 903 | 1751 | 264 | 1278 |
|  | Community | 35.7 | 10.2 | 12.7 | 0.8 | 5.4 | 3.7 | 55.7 | 8.8 |
|  | ED | 45.1 | 70.6 | 78.9 | 84.6 | 82.5 | 58.4 | 27.7 | 72.9 |
|  | Inpatient | 19.2 | 19.2 | 8.4 | 14.6 | 12.1 | 37.9 | 16.7 | 18.3 |

ICD-10-AM = the International Statistical Classification of Diseases and Related Health Problems, Tenth Revision, Australian Modification**;** ED = emergency department

**Table S6** continued

| **Mental health condition (ICD-10-AM)** | **Mental health care setting** | **Schizophrenia and schizoaffective disorders (F20, F25)** | **Specific personality disorders (F60)** | **Bipolar disorders (F30-F31, F34.0)** | **Unspecified mental disorder (F99)** | **Behavioural syndromes associated with physiological disturbances and physical factors (F50-F59)** | **Other personality and behavioural disorders (F61-F69)** | **Early onset behavioural and emotional disorders (F90-F98)** |
| --- | --- | --- | --- | --- | --- | --- | --- | --- |
| Age (%) |  |  |  |  |  |  |  |  |
| 18-24 years | n | 3429 | 6148 | 2412 | 2619 | 2410 | 1149 | 1189 |
|  | Community | 45.5 | 42.2 | 34.9 | 38.7 | 50.2 | 34.9 | 26.7 |
|  | ED | 13.5 | 17.9 | 19.3 | 60.8 | 31.8 | 43.5 | 57.3 |
|  | Inpatient | 41.0 | 39.9 | 45.8 | 0.5 | 28.0 | 21.6 | 16.1 |
| 25-34 years | n | 5109 | 4731 | 3802 | 2696 | 2547 | 1114 | 982 |
|  | Community | 47.5 | 45.2 | 38.1 | 32.6 | 57.5 | 12.9 | 9.9 |
|  | ED | 13.3 | 18.9 | 20.5 | 66.6 | 22.1 | 63.2 | 75.8 |
|  | Inpatient | 39.2 | 35.9 | 41.4 | 0.8 | 20.3 | 23.9 | 14.4 |
| 35-44 years | n | 4165 | 3180 | 3528 | 2292 | 1020 | 825 | 696 |
|  | Community | 49.4 | 44.1 | 39.5 | 34.3 | 53.8 | 15.5 | 9.9 |
|  | ED | 13.7 | 18.4 | 19.2 | 64.8 | 23.2 | 56.4 | 79.7 |
|  | Inpatient | 36.9 | 37.5 | 41.4 | 1.0 | 22.9 | 28.1 | 10.3 |
| 45-54 years | n | 2690 | 1875 | 2887 | 1680 | 254 | 522 | 427 |
|  | Community | 52.8 | 45.9 | 39.6 | 37.9 | 25.6 | 17.1 | 9.6 |
|  | ED | 13.0 | 17.8 | 17.9 | 61.6 | 40.2 | 57.3 | 79.9 |
|  | Inpatient | 34.2 | 36.3 | 42.6 | 0.6 | 34.3 | 25.7 | 10.5 |
| 55-64 years | n | 1344 | 671 | 1685 | 1009 | 182 | 243 | 219 |
|  | Community | 53.6 | 45.8 | 39.3 | 42.2 | 14.8 | 18.1 | 6.4 |
|  | ED | 13.7 | 20.3 | 18.4 | 56.9 | 40.1 | 60.5 | 86.8 |
|  | Inpatient | 32.7 | 34.0 | 42.3 | 0.9 | 45.1 | 21.4 | 6.9 |
| 65-74 years | n | 801 | 264 | 948 | 401 | 149 | 170 | 203 |
|  | Community | 49.8 | 58.0 | 42.8 | 25.7 | 19.5 | 31.2 | 2.5 |
|  | ED | 11.5 | 9.9 | 15.0 | 73.1 | 41.6 | 55.9 | 90.6 |
|  | Inpatient | 38.7 | 32.2 | 42.2 | 1.3 | 38.9 | 12.9 | 6.9 |
| ≥75 years | n | 514 | 177 | 622 | 376 | 183 | 230 | 529 |
|  | Community | 51.8 | 61.0 | 52.4 | 16.0 | 20.8 | 11.3 | 0.8 |
|  | ED | 11.1 | 9.0 | 8.8 | 81.9 | 60.7 | 69.6 | 97.9 |
|  | Inpatient | 37.2 | 29.9 | 38.8 | 2.1 | 18.6 | 19.1 | 1.3 |
| Sex (%) |  |  |  |  |  |  |  |  |
| Male | n | 11,199 | 5745 | 6420 | 5656 | 669 | 2233 | 2461 |
|  | Community | 50.0 | 36.2 | 38.1 | 33.1 | 17.2 | 16.7 | 12.6 |
|  | ED | 13.8 | 26.0 | 19.9 | 66.1 | 50.2 | 58.4 | 77.7 |
|  | Inpatient | 36.2 | 37.8 | 42.0 | 0.8 | 32.6 | 24.9 | 9.7 |
| Female | n | 6852 | 11,299 | 9464 | 5417 | 6076 | 2013 | 1784 |
|  | Community | 47.4 | 48.5 | 39.9 | 37.5 | 53.8 | 25.1 | 13.2 |
|  | ED | 12.5 | 14.1 | 17.6 | 61.7 | 22.0 | 53.0 | 73.0 |
|  | Inpatient | 40.1 | 37.4 | 42.5 | 0.9 | 24.2 | 21.9 | 13.8 |
| Residential remoteness (%) | | | | | | | | |
| Major cities | n | 11,022 | 11,924 | 10,314 | 6245 | 4705 | 3212 | 2914 |
|  | Community | 48.8 | 45.8 | 36.7 | 55.5 | 54.2 | 19.9 | 9.1 |
|  | ED | 12.9 | 16.9 | 18.8 | 44.0 | 22.8 | 57.6 | 79.3 |
|  | Inpatient | 38.4 | 37.3 | 44.5 | 0.6 | 23.0 | 22.5 | 11.6 |
| Inner regional | n | 3145 | 2695 | 2525 | 1275 | 917 | 400 | 639 |
|  | Community | 57.3 | 44.6 | 47.5 | 15.6 | 46.7 | 30.8 | 16.9 |
|  | ED | 10.1 | 22.7 | 16.8 | 82.9 | 28.8 | 33.5 | 74.8 |
|  | Inpatient | 32.6 | 32.7 | 35.7 | 1.5 | 24.5 | 35.8 | 8.3 |
| Outer regional | n | 1229 | 925 | 1227 | 1893 | 423 | 299 | 232 |
|  | Community | 39.5 | 39.4 | 40.5 | 3.1 | 25.3 | 20.7 | 22.8 |
|  | ED | 16.8 | 16.2 | 17.7 | 96.5 | 36.2 | 64.6 | 61.6 |
|  | Inpatient | 43.6 | 44.4 | 41.8 | 0.4 | 38.5 | 14.7 | 15.5 |
| Remote | n | 764 | 505 | 696 | 676 | 312 | 96 | 122 |
|  | Community | 45.9 | 39.6 | 41.2 | 4.9 | 51.6 | 22.9 | 49.2 |
|  | ED | 17.5 | 13.3 | 16.1 | 93.8 | 17.3 | 50.0 | 32.8 |
|  | Inpatient | 36.5 | 47.1 | 42.7 | 1.3 | 31.1 | 27.1 | 18.0 |
| Very remote | n | 971 | 391 | 560 | 559 | 259 | 63 | 106 |
|  | Community | 56.1 | 47.1 | 48.4 | 7.5 | 29.0 | 31.8 | 46.2 |
|  | ED | 9.0 | 14.1 | 12.7 | 90.3 | 34.8 | 36.5 | 26.4 |
|  | Inpatient | 34.9 | 38.9 | 38.9 | 2.2 | 36.3 | 31.8 | 27.4 |
| Missing | n | 921 | 606 | 562 | 425 | 129 | 183 | 232 |
|  | Community | 31.2 | 25.7 | 31.9 | 24.9 | 46.5 | 10.9 | 5.6 |
|  | ED | 25.5 | 31.5 | 32.6 | 73.9 | 31.0 | 66.1 | 91.8 |
|  | Inpatient | 43.3 | 42.7 | 35.6 | 1.2 | 22.5 | 23.0 | 2.6 |
| Socioeconomic status (%) | | | | | | | | |
| Quintile 1 | n | 703 | 326 | 334 | 711 | 159 | 67 | 79 |
|  | Community | 50.9 | 45.7 | 43.1 | 2.5 | 16.4 | 17.9 | 41.8 |
|  | ED | 12.4 | 16.9 | 13.8 | 95.6 | 40.9 | 49.3 | 38.0 |
|  | Inpatient | 36.7 | 37.4 | 43.1 | 1.8 | 42.8 | 32.8 | 20.3 |
| Quintile 2 | n | 902 | 777 | 621 | 1007 | 262 | 176 | 150 |
|  | Community | 46.0 | 43.2 | 43.3 | 4.8 | 27.1 | 21.0 | 17.3 |
|  | ED | 12.8 | 18.2 | 16.8 | 94.5 | 43.5 | 52.8 | 66.7 |
|  | Inpatient | 41.2 | 38.6 | 39.9 | 0.7 | 29.4 | 26.1 | 16.0 |
| Quintile 3 | n | 2297 | 2591 | 2042 | 1662 | 715 | 603 | 583 |
|  | Community | 49.2 | 51.0 | 45.7 | 16.3 | 40.8 | 20.9 | 17.5 |
|  | ED | 13.1 | 14.6 | 15.2 | 83.3 | 32.6 | 59.4 | 71.4 |
|  | Inpatient | 37.7 | 34.5 | 39.1 | 0.5 | 26.6 | 19.7 | 11.2 |
| Quintile 4 | n | 5163 | 5363 | 4557 | 3488 | 1806 | 1641 | 1497 |
|  | Community | 48.9 | 45.0 | 39.0 | 36.2 | 48.1 | 16.1 | 12.3 |
|  | ED | 17.5 | 23.9 | 24.9 | 63.1 | 31.7 | 69.7 | 79.0 |
|  | Inpatient | 33.6 | 31.1 | 36.1 | 0.7 | 20.3 | 14.2 | 8.8 |
| Quintile 5 | n | 8066 | 7383 | 7768 | 3780 | 3674 | 1583 | 1704 |
|  | Community | 51.3 | 43.2 | 37.5 | 58.1 | 56.2 | 26.9 | 11.1 |
|  | ED | 9.4 | 14.1 | 15.0 | 41.0 | 17.7 | 39.2 | 74.7 |
|  | Inpatient | 39.3 | 42.7 | 47.5 | 0.9 | 26.1 | 33.9 | 14.3 |
| Missing | n | 921 | 606 | 562 | 425 | 129 | 183 | 232 |
|  | Community | 31.2 | 25.7 | 31.9 | 24.9 | 46.5 | 10.9 | 5.6 |
|  | ED | 25.5 | 31.5 | 32.6 | 73.9 | 31.0 | 66.1 | 91.8 |
|  | Inpatient | 43.3 | 42.7 | 35.6 | 1.2 | 22.5 | 23.0 | 2.6 |

ICD-10-AM = the International Statistical Classification of Diseases and Related Health Problems, Tenth Revision, Australian Modification**;** ED = emergency department;

**Table S6** Continued

| **Mental health condition (ICD-10-AM)** | **Mental health care setting** | **Somatoform disorders (F45)** | **Dissociative disorders (F44)** | **Obsessive compulsive disorders (F42)** | **Other affective disorders (F34 (excluding 34.0, 34.1), F38-F39)** | **Other neurotic disorders (F48)** | **Disorders of psychological development (F80-F89)** | **Mental retardation (F70-F79)** |
| --- | --- | --- | --- | --- | --- | --- | --- | --- |
| Age (%) |  |  |  |  |  |  |  |  |
| 18-24 years | n | 583 | 524 | 563 | 341 | 262 | 455 | 163 |
|  | Community | 10.0 | 5.3 | 65.4 | 33.7 | 3.8 | 59.8 | 58.3 |
|  | ED | 72.7 | 37.8 | 11.2 | 27.3 | 92.4 | 5.3 | 4.3 |
|  | Inpatient | 17.3 | 56.9 | 23.5 | 39.0 | 3.8 | 35.0 | 37.4 |
| 25-34 years | n | 606 | 638 | 592 | 358 | 276 | 204 | 116 |
|  | Community | 10.4 | 3.5 | 63.2 | 45.0 | 1.8 | 55.9 | 47.4 |
|  | ED | 68.8 | 38.7 | 9.8 | 23.5 | 92.0 | 14.2 | 7.8 |
|  | Inpatient | 20.8 | 57.8 | 27.0 | 31.6 | 6.2 | 29.9 | 44.8 |
| 35-44 years | n | 553 | 594 | 327 | 298 | 247 | 80 | 101 |
|  | Community | 10.3 | 1.5 | 63.3 | 45.3 | 3.6 | 43.8 | 46.5 |
|  | ED | 57.0 | 30.3 | 9.8 | 23.5 | 93.5 | 16.3 | 3.0 |
|  | Inpatient | 32.7 | 68.2 | 26.9 | 31.2 | 2.8 | 40.0 | 50.5 |
| 45-54 years | n | 496 | 499 | 195 | 230 | 153 | 52 | 92 |
|  | Community | 6.5 | 5.2 | 63.1 | 35.2 | 3.9 | 53.9 | 43.5 |
|  | ED | 47.4 | 23.9 | 7.2 | 30.4 | 85.6 | 17.3 | 2.2 |
|  | Inpatient | 46.2 | 70.9 | 29.7 | 34.4 | 10.5 | 28.9 | 54.4 |
| 55-64 years | n | 347 | 308 | 118 | 128 | 97 | 27 | 53 |
|  | Community | 8.7 | 5.2 | 57.6 | 38.3 | 3.1 | 40.7 | 43.4 |
|  | ED | 34.9 | 22.4 | 13.6 | 25.8 | 89.7 | 22.2 | 5.7 |
|  | Inpatient | 56.5 | 72.4 | 28.8 | 35.9 | 7.2 | 37.0 | 50.9 |
| 65-74 years | n | 217 | 160 | 69 | 65 | 42 | 14 | 51 |
|  | Community | 11.1 | 5.0 | 71.0 | 49.2 | 4.8 | 71.4 | 58.8 |
|  | ED | 25.8 | 23.1 | 4.4 | 21.5 | 83.3 | 0 | 5.9 |
|  | Inpatient | 63.1 | 71.9 | 24.6 | 29.2 | 11.9 | 28.6 | 35.3 |
| ≥75 years | n | 201 | 127 | 53 | 68 | 59 | 5 | 15 |
|  | Community | 8.5 | 3.9 | 71.7 | 51.5 | 1.7 | 60.0 | 53.3 |
|  | ED | 33.3 | 19.7 | 7.6 | 16.2 | 84.8 | 20.0 | 13.3 |
|  | Inpatient | 58.2 | 76.4 | 20.8 | 32.4 | 13.6 | 20.0 | 33.3 |
| Sex (%) |  |  |  |  |  |  |  |  |
| Male | n | 1014 | 821 | 794 | 614 | 429 | 604 | 298 |
|  | Community | 9.9 | 3.3 | 63.7 | 40.2 | 3.5 | 58.0 | 49.3 |
|  | ED | 53.9 | 32.0 | 11.0 | 28.8 | 92.1 | 7.3 | 4.0 |
|  | Inpatient | 36.3 | 64.7 | 25.3 | 30.9 | 4.4 | 34.8 | 46.6 |
| Female | n | 1989 | 2029 | 1123 | 874 | 707 | 232 | 293 |
|  | Community | 9.1 | 4.3 | 64.2 | 41.3 | 3.0 | 52.6 | 51.5 |
|  | ED | 54.8 | 30.2 | 9.2 | 22.7 | 89.8 | 16.4 | 5.8 |
|  | Inpatient | 36.2 | 65.6 | 26.6 | 36.0 | 7.2 | 31.0 | 42.7 |
| Residential remoteness (%)50 | | | | | | | | |
| Major cities | N | 1988 | 1938 | 1301 | 838 | 642 | 585 | 370 |
|  | Community | 10.1 | 4.0 | 61.3 | 42.8 | 3.6 | 54.5 | 52.2 |
|  | ED | 53.2 | 32.4 | 9.5 | 16.5 | 89.3 | 7.0 | 2.2 |
|  | Inpatient | 36.8 | 63.7 | 29.2 | 40.7 | 7.2 | 38.5 | 45.7 |
| Inner regional | n | 557 | 376 | 319 | 193 | 389 | 117 | 91 |
|  | Community | 8.6 | 4.3 | 67.4 | 60.6 | 2.1 | 67.5 | 57.1 |
|  | ED | 65.0 | 33.2 | 14.1 | 9.8 | 95.6 | 9.4 | 3.3 |
|  | Inpatient | 26.4 | 62.5 | 18.5 | 29.5 | 2.3 | 23.1 | 39.6 |
| Outer regional | n | 179 | 230 | 127 | 192 | 28 | 69 | 50 |
|  | Community | 7.3 | 4.4 | 64.6 | 32.8 | 10.7 | 50.7 | 48.0 |
|  | ED | 39.7 | 23.5 | 10.2 | 44.8 | 67.9 | 24.6 | 20.0 |
|  | Inpatient | 53.1 | 72.2 | 25.2 | 22.4 | 21.4 | 24.6 | 32.0 |
| Remote | n | 130 | 120 | 76 | 104 | 10 | 25 | 34 |
|  | Community | 5.4 | 5.0 | 76.3 | 26.0 | 0 | 72.0 | 26.5 |
|  | ED | 50.0 | 18.3 | 1.3 | 47.1 | 60.0 | 8.0 | 8.8 |
|  | Inpatient | 44.6 | 76.7 | 22.4 | 26.9 | 40.0 | 20.0 | 64.7 |
| Very remote | n | 70 | 100 | 61 | 128 | 14 | 25 | 19 |
|  | Community | 7.1 | 2.0 | 82.0 | 21.9 | 14.3 | 52.0 | 42.1 |
|  | ED | 37.1 | 11.0 | 1.6 | 60.9 | 64.3 | 32.0 | 21.1 |
|  | Inpatient | 55.7 | 87.0 | 16.4 | 17.2 | 21.4 | 16.0 | 36.8 |
| Missing | n | 79 | 86 | 33 | 33 | 53 | 16 | 27 |
|  | Community | 10.1 | 3.5 | 72.7 | 42.4 | 0 | 56.3 | 44.4 |
|  | ED | 68.4 | 41.9 | 21.2 | 15.2 | 96.2 | 18.8 | 3.7 |
|  | Inpatient | 21.5 | 54.7 | 6.1 | 42.4 | 3.8 | 25.0 | 51.9 |
| Socioeconomic status (%) | | | | | | | | |
| Quintile 1 | n | 57 | 80 | 34 | 96 | 11 | 22 | 20 |
|  | Community | 1.8 | 0 | 82.4 | 27.1 | 0 | 68.2 | 35.0 |
|  | ED | 47.4 | 12.5 | 0 | 59.4 | 81.8 | 13.6 | 20.0 |
|  | Inpatient | 50.9 | 87.5 | 17.7 | 13.5 | 18.2 | 18.2 | 45.0 |
| Quintile 2 | n | 117 | 138 | 64 | 107 | 52 | 49 | 32 |
|  | Community | 7.7 | 4.4 | 60.9 | 34.6 | 7.7 | 61.2 | 53.1 |
|  | ED | 46.2 | 21.7 | 14.1 | 43.9 | 80.8 | 30.6 | 12.5 |
|  | Inpatient | 46.2 | 73.9 | 25.0 | 21.5 | 11.5 | 8.2 | 34.4 |
| Quintile 3 | n | 397 | 428 | 257 | 320 | 154 | 114 | 97 |
|  | Community | 8.1 | 5.6 | 65.0 | 39.7 | 3.3 | 61.4 | 56.7 |
|  | ED | 56.2 | 29.4 | 8.6 | 42.8 | 90.9 | 9.7 | 7.2 |
|  | Inpatient | 35.8 | 65.0 | 26.5 | 17.5 | 5.8 | 29.0 | 36.1 |
| Quintile 4 | n | 1090 | 900 | 556 | 396 | 492 | 246 | 168 |
|  | Community | 7.7 | 3.7 | 66.2 | 45.7 | 2.2 | 57.3 | 47.6 |
|  | ED | 63.9 | 37.2 | 14.8 | 18.4 | 94.5 | 9.4 | 6.6 |
|  | Inpatient | 28.4 | 59.1 | 19.1 | 35.9 | 3.3 | 33.3 | 45.8 |
| Quintile 5 | n | 1263 | 1218 | 973 | 536 | 374 | 390 | 247 |
|  | Community | 11.6 | 3.9 | 61.8 | 41.6 | 4.3 | 53.3 | 51.4 |
|  | ED | 46.0 | 27.8 | 7.2 | 10.5 | 86.4 | 6.9 | 0.8 |
|  | Inpatient | 42.4 | 68.3 | 31.0 | 48.0 | 9.4 | 39.7 | 47.8 |
| Missing | n | 79 | 86 | 33 | 33 | 53 | 16 | 27 |
|  | Community | 10.1 | 3.5 | 72.7 | 42.4 | 0 | 56.3 | 44.4 |
|  | ED | 68.4 | 41.9 | 21.2 | 15.2 | 96.2 | 18.8 | 3.7 |
|  | Inpatient | 21.5 | 54.7 | 6.1 | 42.4 | 3.8 | 25.0 | 51.9 |

ICD-10-AM = the International Statistical Classification of Diseases and Related Health Problems, Tenth Revision, Australian Modification**;** ED = emergency department;

**
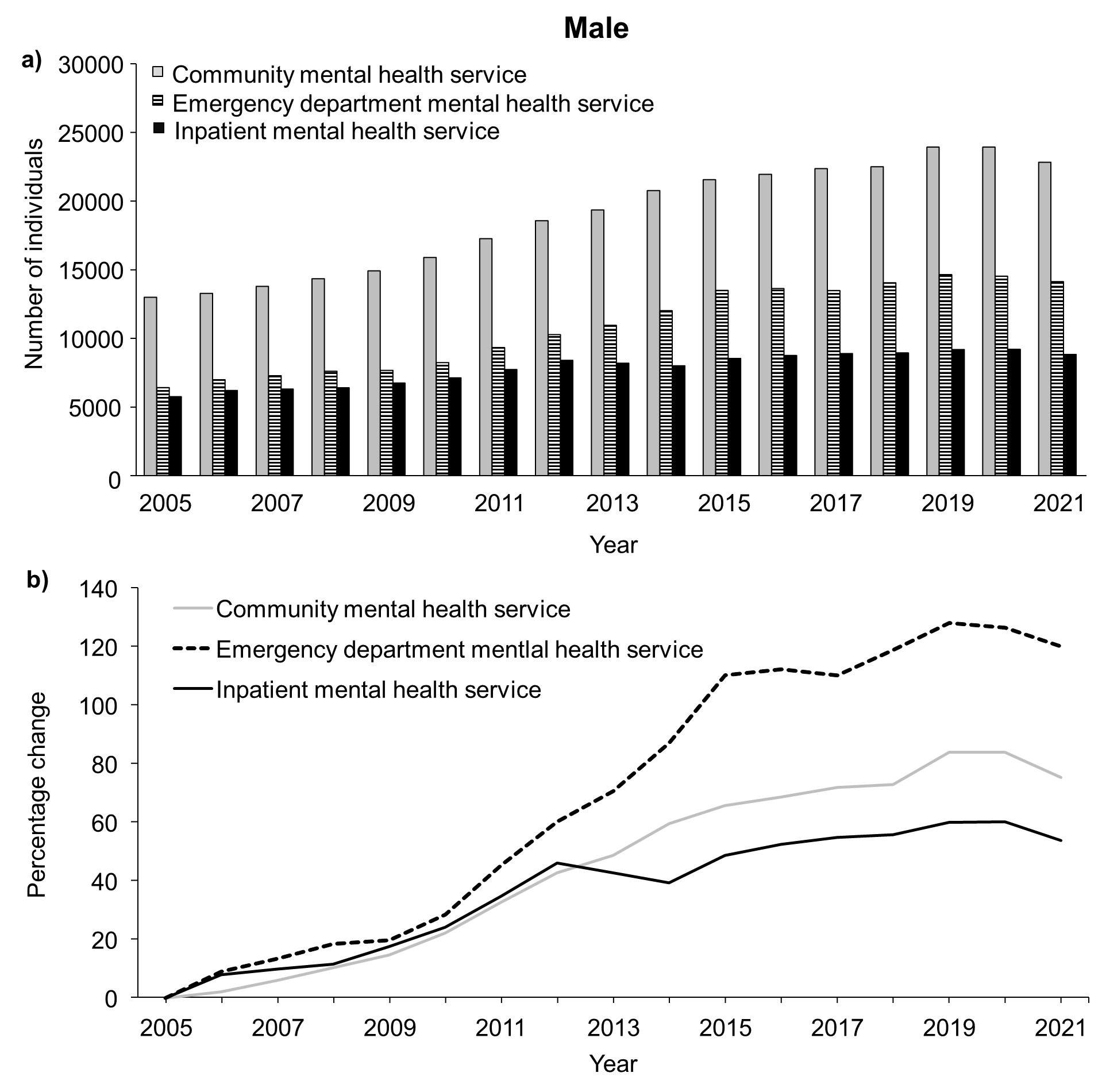

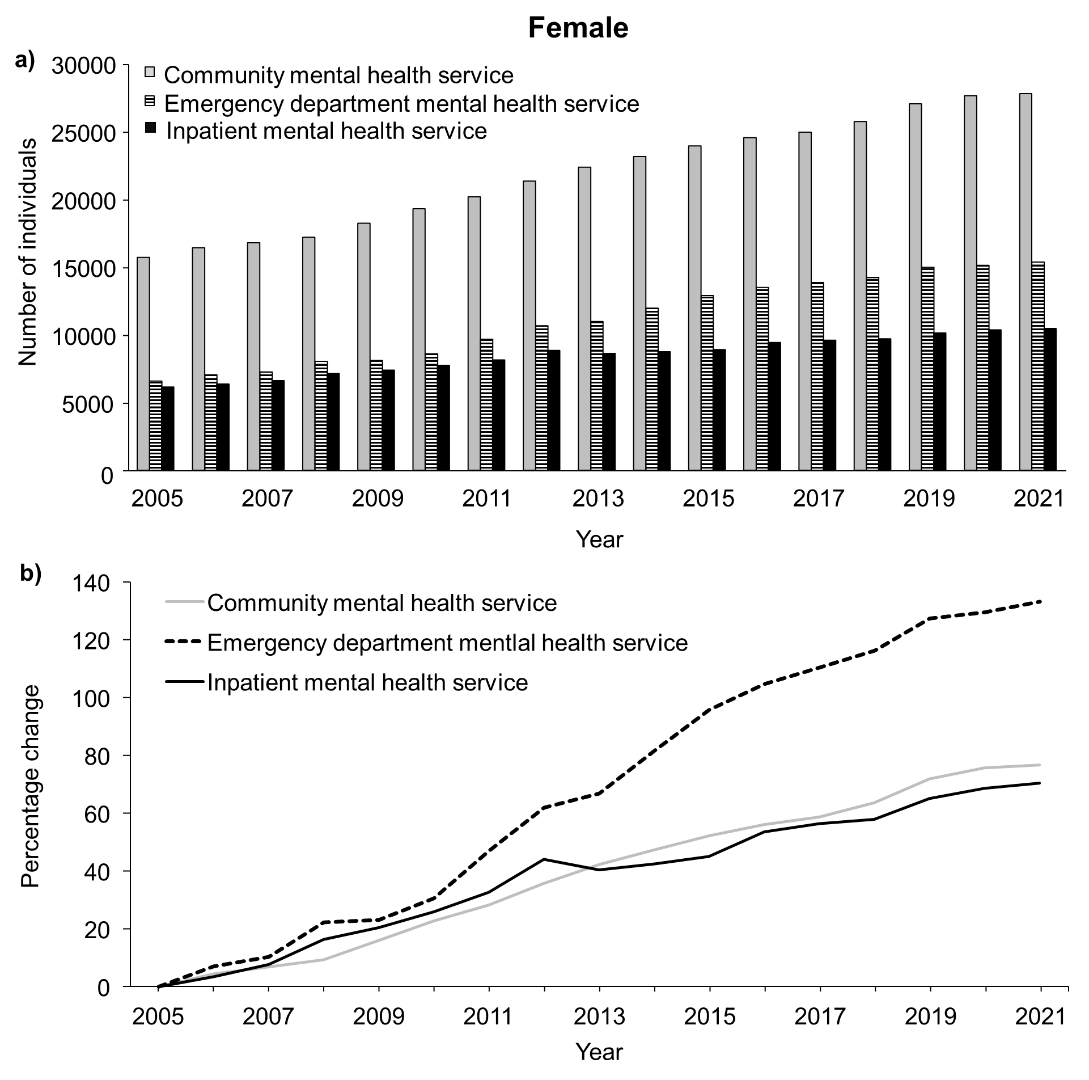
**

**Fig S1** a) Number of individuals, and b) change in number of individuals relative to 2005, who accessed state-funded mental health services by care setting and sex

**
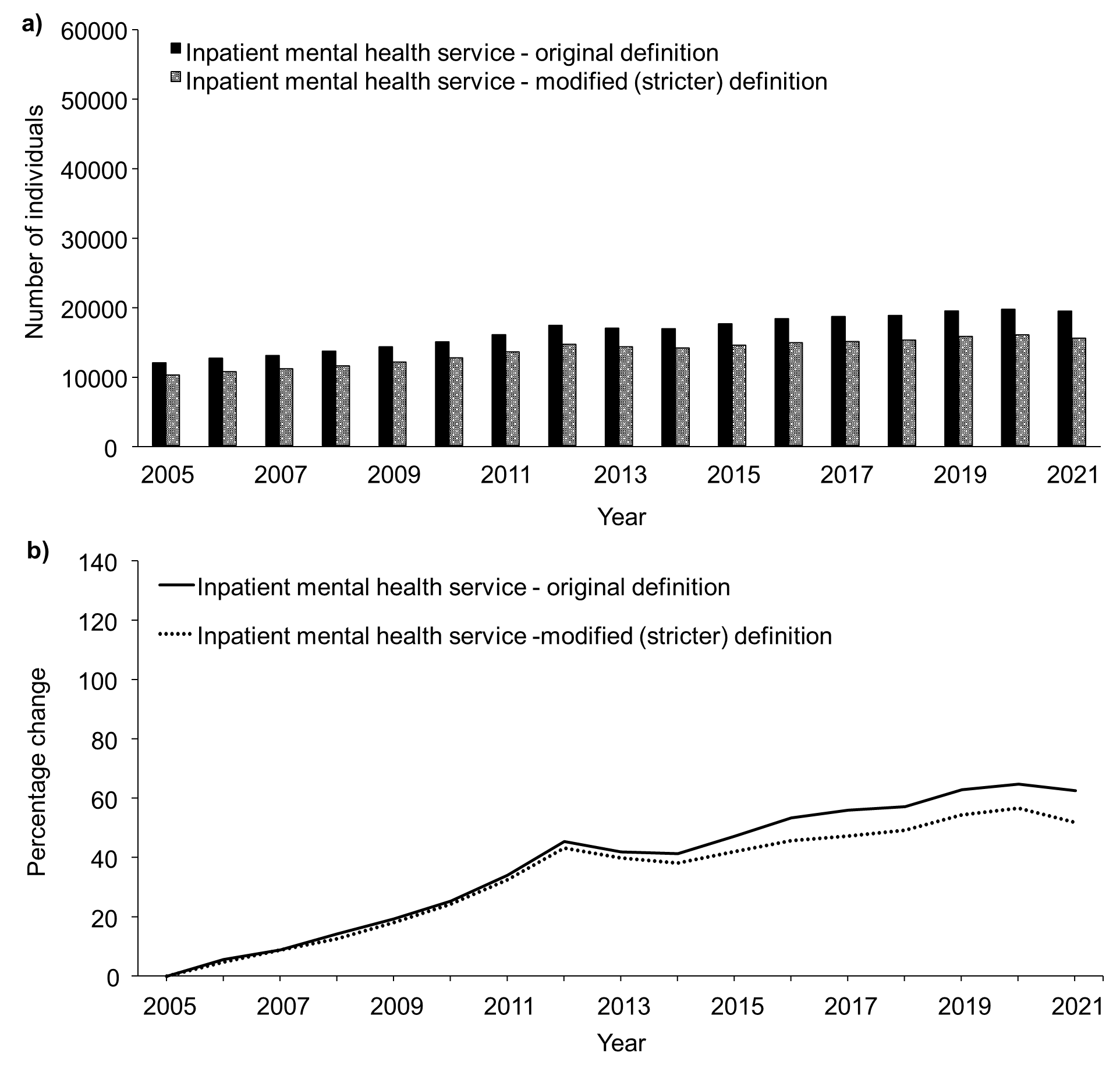
**

**Fig S2** a) Number of individuals, and b) change in number of individuals relative to 2005 who accessed inpatient mental health services by definition of inpatient mental health service

**
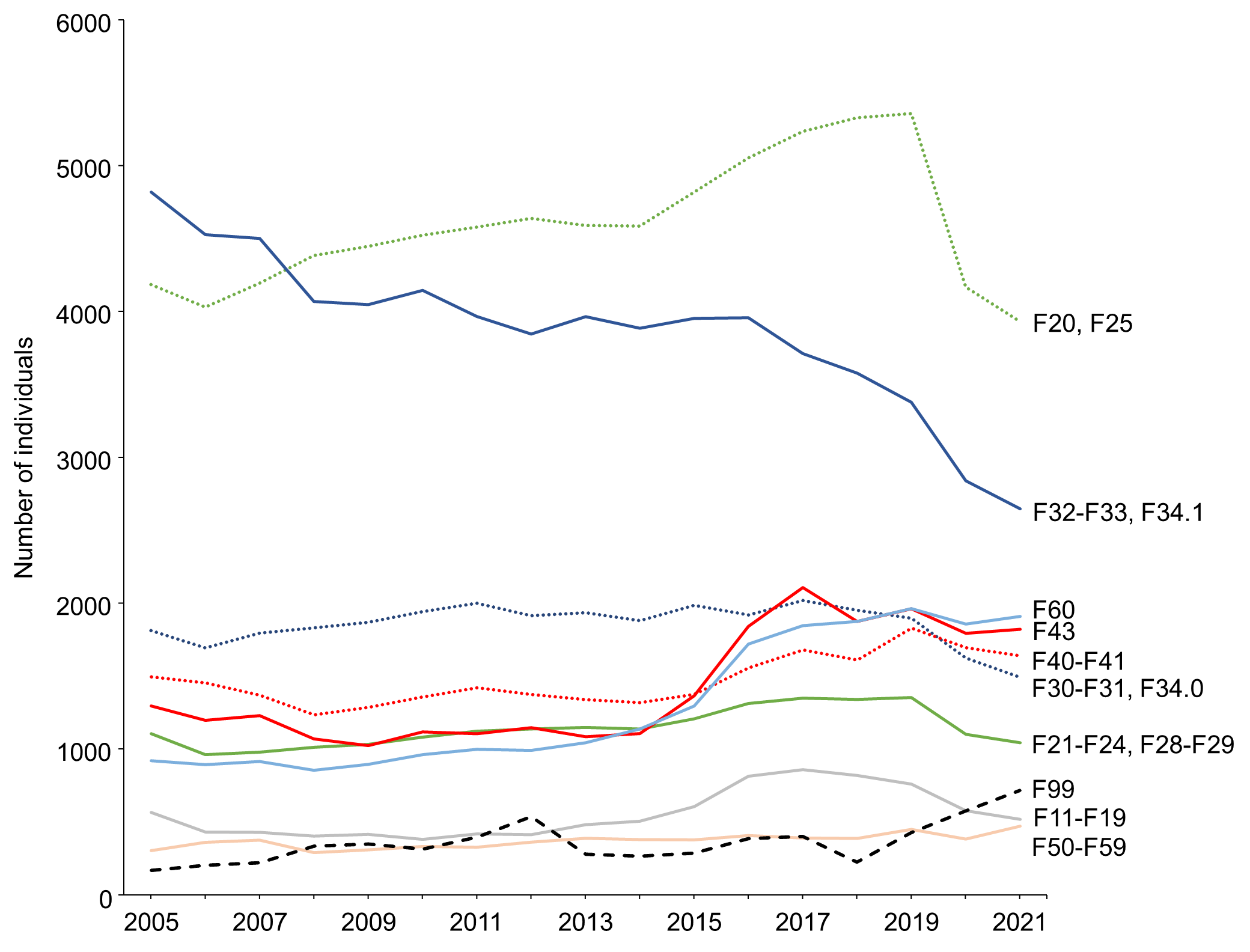
**

**Fig S3** Number of individuals who accessed state-funded mental health services by mental health conditions for the ten conditions with the most individuals with at least one community mental health service contact

F11-19 = drug use disorders; F20, F25 = schizophrenia and schizoaffective disorders; F30-F31, F34.0 = bipolar disorders; F32-F33, F34.1 = major depressive disorders; F40-F41 anxiety disorders; F43 = reaction to severe stress, and adjustment disorders; F50-F59 = behavioural syndromes associated with physiological disturbances and physical factors; F60 = specific personality disorders; F99 = unspecified mental disorder;

**
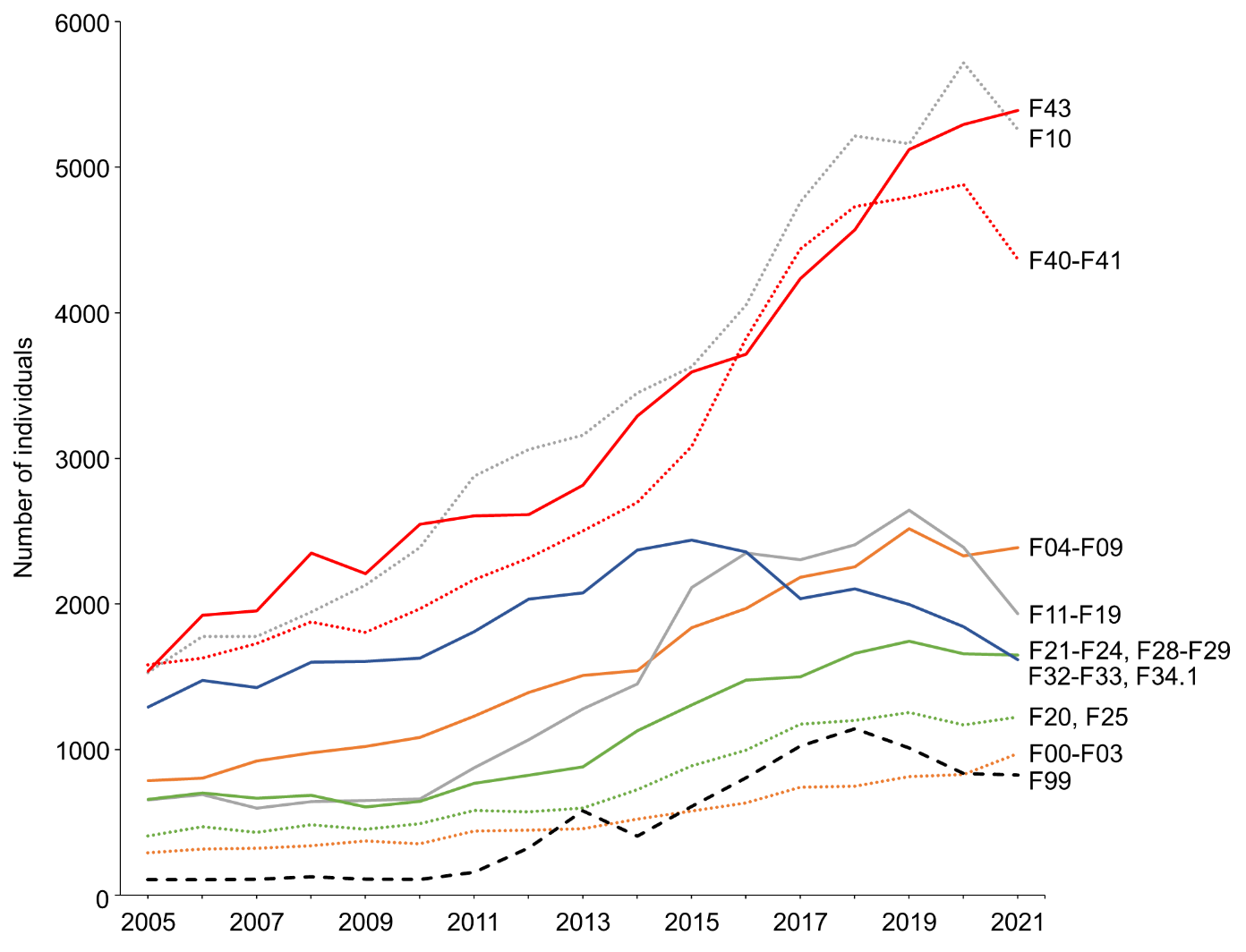
**

**Fig S4** Number of individuals who accessed state-funded mental health services by mental health conditions for the ten conditions with the most individuals with at least one emergency department mental health service contact

F00-F03 = dementia; F04-F09 = organic mental disorders not including dementia; F10 = alcohol disorder; F11-F19 = drug use disorders; F20, F25 = schizophrenia and schizoaffective disorders; F21-F24, F28-F29 = schizotypal and delusional disorders; F32-F33, F34.1 = major depressive disorders; F40-F41 = anxiety disorders; F43 = reaction to severe stress, and adjustment disorders; F99 = unspecified mental disorders;

**
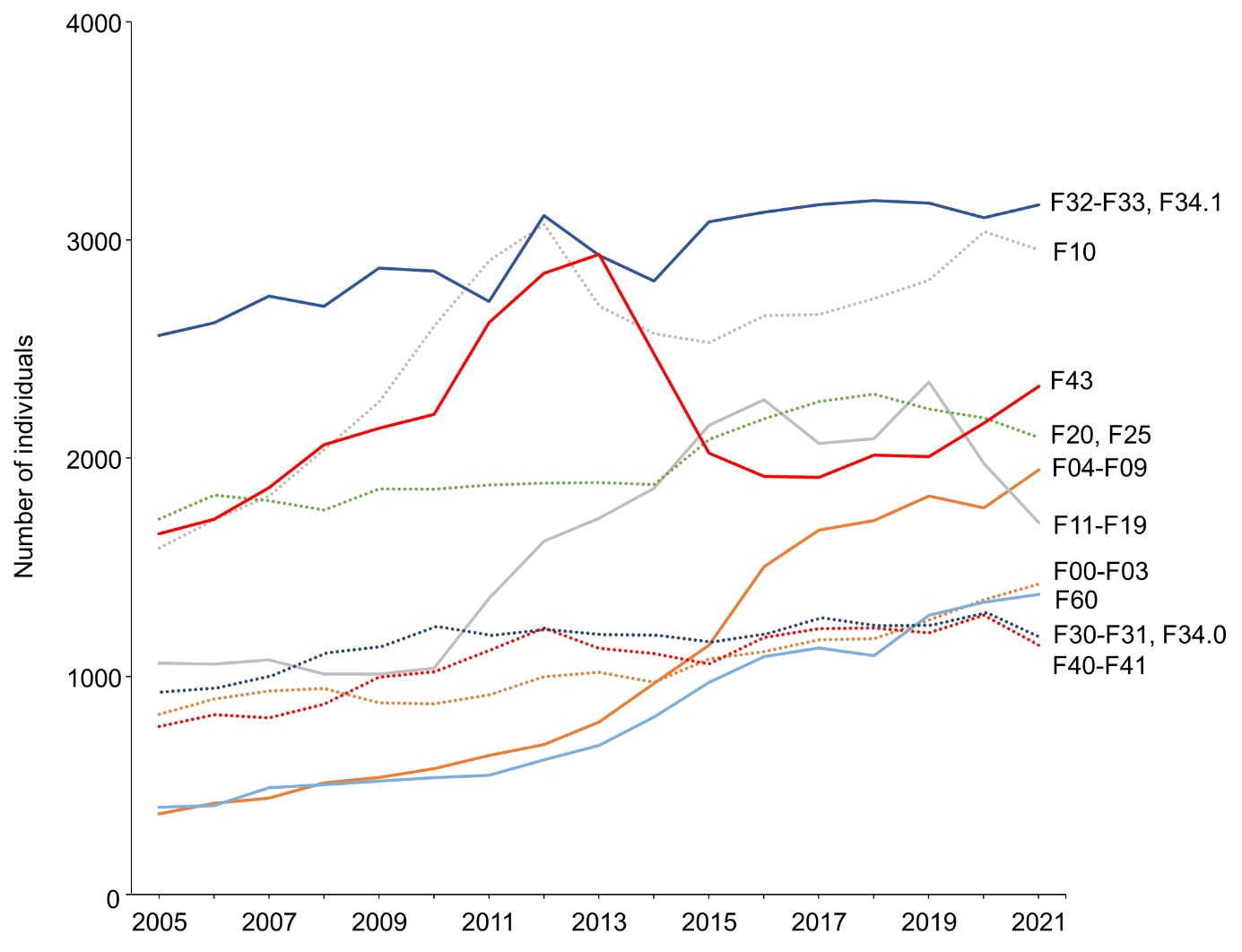
**

**Fig S5** Number of individuals who accessed state-funded mental health services by mental health conditions for the ten conditions with the most individuals with at least one inpatient mental health service contact

F00-F03 = dementia; F04-F09 = organic mental disorders not including dementia; F10 = alcohol disorder; F11-F19 = drug use disorders; F20, F25 = schizophrenia and schizoaffective disorders; F30-F31, F34.0 = bipolar disorders; F32-F33, F34.1 = major depressive disorders; F40-F41 = anxiety disorders; F43 = reaction to severe stress, and adjustment disorders; F60 = specific personality disorders;

**
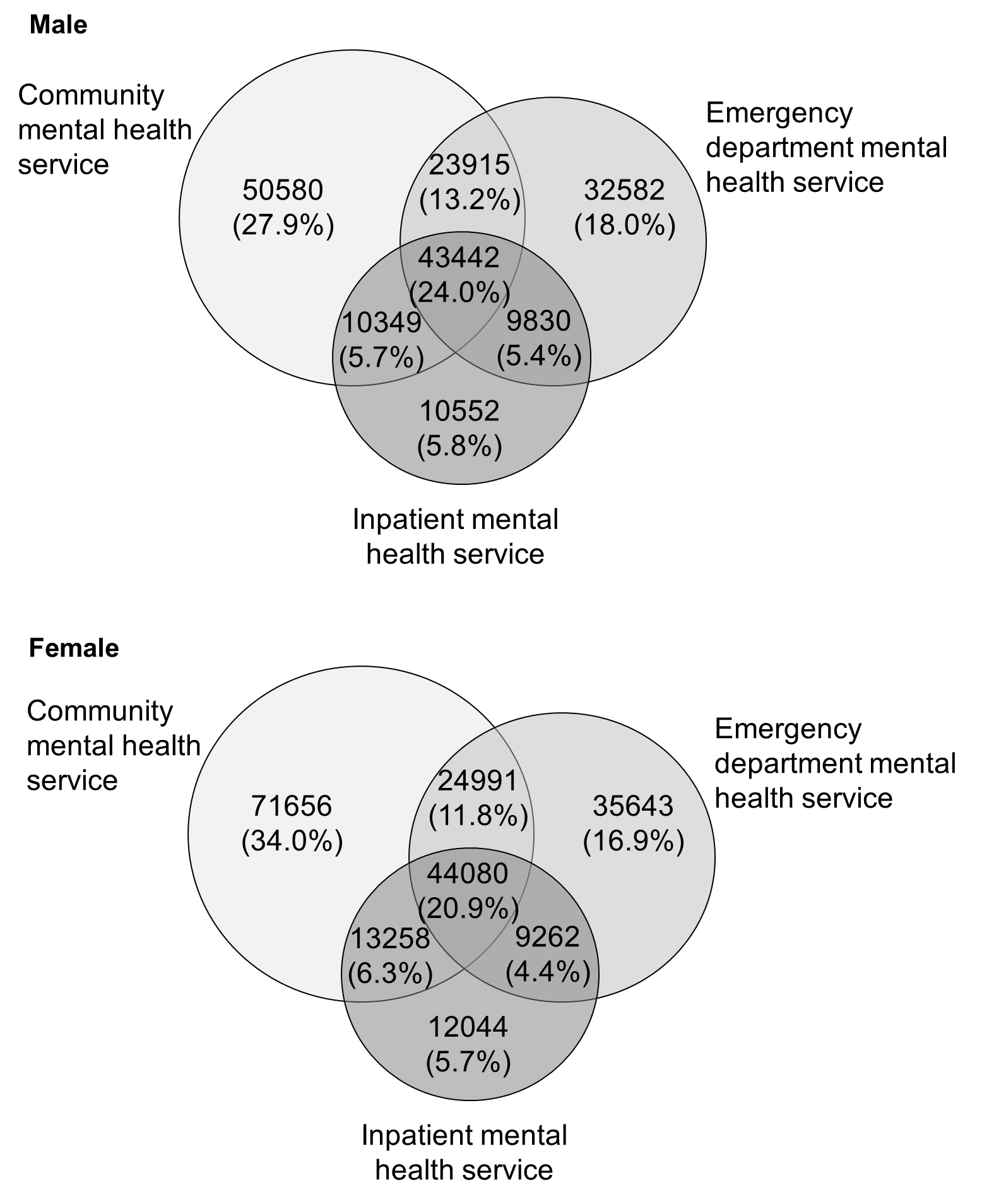
**

**Fig S6** Pattern of access of state-funded mental health services for the period 2005-2021 by sex

**
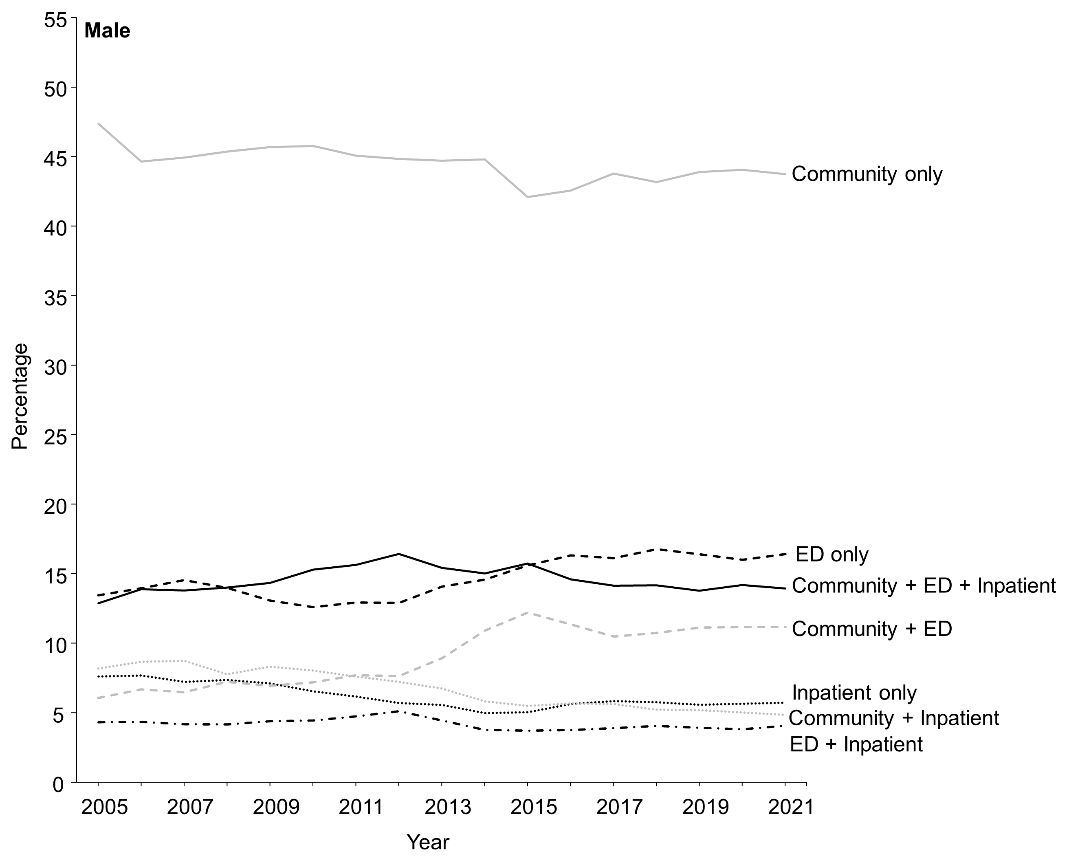

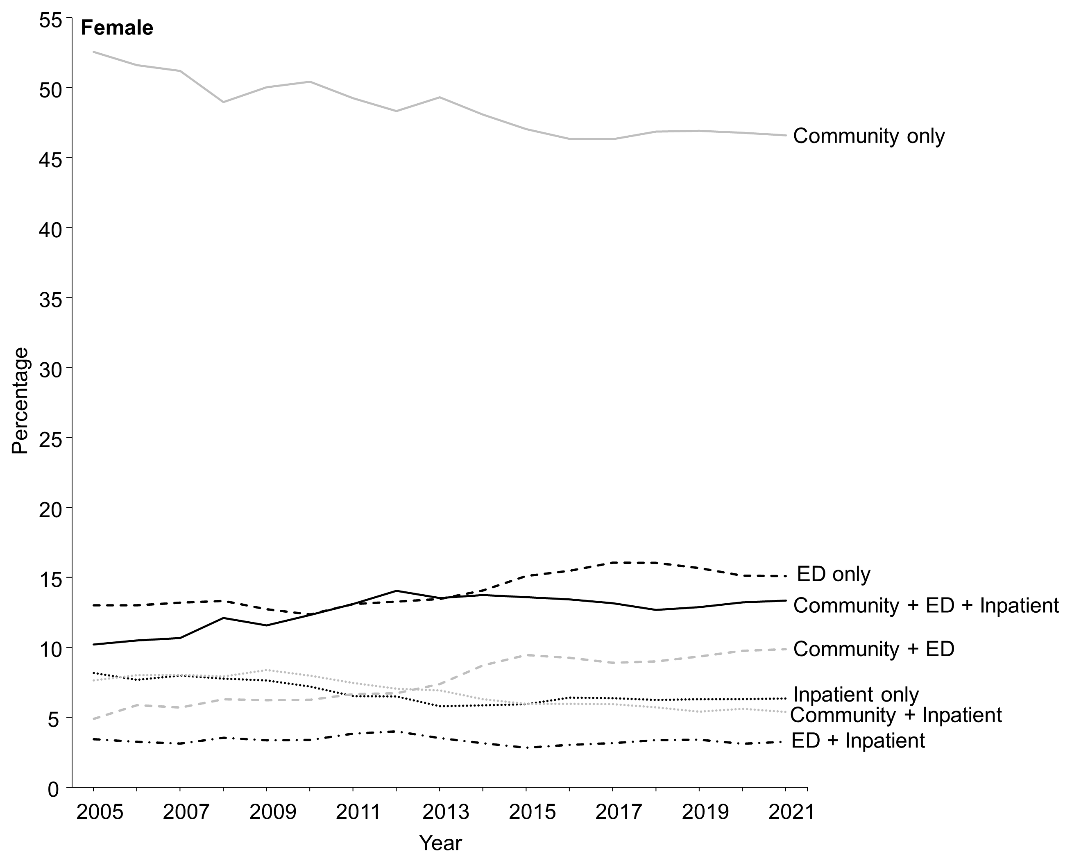
**

**Fig S7** Pattern of access of state-funded mental health services (community, emergency department (ED), inpatient) by year and sex
